# An emerging coastal wetland management dilemma between mangrove expansion and shorebird conservation

**DOI:** 10.1111/cobi.13905

**Published:** 2022-06-08

**Authors:** Chi‐Yeung Choi, Hui Xiao, Mingming Jia, Micha V. Jackson, Yi‐Chien Lai, Nicholas J. Murray, Luke Gibson, Richard A. Fuller

**Affiliations:** ^1^ School of Environmental Science and Engineering Southern University of Science and Technology Shenzhen China; ^2^ School of Biological Sciences The University of Queensland Brisbane Queensland Australia; ^3^ School of Life & Environmental Sciences Deakin University Burwood Victoria Australia; ^4^ Key Laboratory of Wetland Ecology and Environment Northeast Institute of Geography and Agroecology Chinese Academy of Sciences Changchun China; ^5^ Department of Environmental Science and Engineering Tunghai University Taichung Taiwan; ^6^ College of Science and Engineering James Cook University Townsville Queensland Australia

**Keywords:** blue carbon, East Asian‐Australasian Flyway, saltmarsh, restoration, tidal flat, wader, carbono azul, limícola, marisma, planicie intermareal, restauración, Ruta de Asia Oriental‐Australasia, 蓝碳;东亚‐澳大利西亚迁飞区;盐沼;修复;滩涂;涉禽

## Abstract

Coastal wetlands around the world have been degraded by human activities. Global declines in the extent of important coastal wetlands, including mangroves, salt marshes, and tidal flats, necessitate mitigation and restoration efforts. However, some well‐meaning management actions, particularly mangrove afforestation, can inadvertently cause further loss and degradation of other habitats if these actions are not planned carefully. In particular, there is a potential conflict between mangrove and shorebird conservation because mangrove afforestation and restoration may occur at the expense of bare tidal flats, which form the main foraging habitats for threatened shorebirds and support other coastal organisms. We examined several case studies that illustrate the trade‐off between mangrove restoration and bare tidal flat maintenance. To investigate whether these examples reflect an emerging broad‐scale problem, we used satellite imagery to quantify the change in mangrove habitat extent in 22 important shorebird areas in mainland China from 2000 to 2015.The extent of tidal flat across all sites declined significantly (*p* < 0.01, *n* = 22) while among sites with mangroves present, the extent of mangroves expanded significantly (*p* < 0.01, *n* = 14). Our results suggest mangrove expansion and tidal flat loss have considerably reduced shorebird habitat in 8 of these sites. To improve the overall conservation outcome, we devised a decision tree for addressing the dilemma. Important factors to consider include whether the area of interest is of importance to shorebirds and what the potential impacts of mangrove expansion are; what the value of the proposed mangrove ecosystem is compared with the existing ecosystem; and that a conflict‐resolution process will be needed if the choices are very similar. With careful consideration of alternative management strategies, decision makers can ensure that the conservation of mangroves does not imperil migratory shorebirds.

## Abstract 2

*Article Impact Statement*: A structural decision‐making framework is proposed to resolve the wetland management dilemma between mangrove and shorebird conservation.

## Abstract (es)

Un Dilema Emergente en la Gestión de Humedales Costeros entre la Expansión de los Manglares y la Conservación de Aves Playeras

**Resumen:**

Los humedales costeros han sido degradados por las actividades humanas a nivel mundial. La declinación global de la extensión de importantes humedales costeros, incluyendo a los manglares, las marismas y las planicies intermareales, requiere mitigación y esfuerzos de restauración. Sin embargo, algunas gestiones bien intencionadas, en especial la reforestación de los manglares, pueden causar inadvertidamente mayores pérdidas y degradaciones si estas acciones no se planean meticulosamente. Particularmente, existe un conflicto potencial entre la conservación de los manglares y las aves playeras pues la reforestación y restauración de los manglares puede perjudicar a las planicies intermareales, que son los principales hábitats de alimentación para aves playeras amenazadas y también sustentan a otros organismos costeros. Analizamos varios estudios de caso que representan la compensación entre la restauración de los manglares y el mantenimiento de las planicies intermareales. Para investigar si estos ejemplos reflejaban un problema emergente a escala amplia, usamos imágenes satelitales para desarrollar una cuantificación detallada del cambio en la extensión del manglar en 22 áreas relevantes de aves playeras en la China continental entre el 2000 y el 2015. La extensión de los manglares en todos los sitios se expandió significativamente (*p* < 0.01, *n* = 14), mientras que la extensión de las planicies intermareales declinó considerablemente (*p* < 0.01, *n* = 22). Nuestros resultados sugieren que la expansión de los manglares y la reducción de las planicies intermareales han reducido notablemente el hábitat de las aves playeras en ocho de los 22 sitios. Para mejorar la conservación de los manglares y las aves playeras, diseñamos un árbol de decisiones para abordar este dilema. Los factores importantes para considerar incluyen si el área de interés es importante para las aves y cuáles son los impactos potenciales de la expansión del manglar; cuál es el valor del ecosistema propuesto en comparación con el ecosistema existente; y que un proceso de resolución de conflictos será necesario si las opciones son muy similares. Mediante la consideración meticulosa de las estrategias alternativas de manejo, los órganos decisorios pueden asegurar que la conservación de los manglares no ponga en peligro a las aves playeras migratorias.

## 滨海湿地管理的新窘境 – 红树林扩张与鸻鹬类水鸟保护

摘要: 在人类活动的影响下，全球的滨海湿地都在退化, 包括红树林、盐沼和光滩在内的重要滨海湿地类型的面积都在下降, 减缓这一情况并修复受损湿地变得尤为迫切。然而, 一些出发点很好的管理行动, 尤其是红树林造林, 如果没有周详谨慎的计划, 就有可能会在无意间导致更多的滨海湿地的丧失和退化。红树林与鸻鹬类保护之间是存在着潜在的矛盾, 因为红树林造林或恢复可能会以牺牲潮间带光滩区域为代价, 就是一些受胁滨海迁徙鸻鹬类水鸟以及其它滨海物种的主要觅食生境。本文列举多个研究案例来说明红树林恢复和光滩保护之间的权衡问题。为了了解这些案例是否代表了一个新出现的普遍现象, 我们利用卫星图片详细量化了中国大陆22个重要的鸻鹬类栖息地内红树林生境的面积在在2000和2015年间的变化。研究结果表明红树林面积在其有所分布的地点均出现显著增长(*p* < 0.01, *n* = 14)，而光滩面积则显著减少(*p* < 0.01, *n* = 22)。我们的结果同时显示, 红树林扩张和光滩丧失导致了其中 8 个地点的鸻鹬类栖息地面积大大减少。我们设计了一个决策树来解决红树林恢复和鸻鹬类保护之间的矛盾, 当中重要的考虑因子包括所关注的区域对鸻鹬类的重要性;红树林扩张的潜在影响;与现有的生态系统相比, 拟议的红树林生态系统的价值是如何。如果双方的生态系统价值相近难以选择, 则需要一个解决冲突的权衡过程。通过仔细地考虑替代管理策略, 决策者方可确保红树林的保护和修复不会危及迁徙鸻鹬类水鸟。

## INTRODUCTION

### Current state and trajectory of mangrove habitat

Mangroves occur in the intertidal zone, where they are regularly inundated by seawater, and are most abundant and diverse in the tropics (Giri et al., 2011). Mangrove ecosystems provide important ecosystem services, including shoreline protection (Sun & Carson, 2020), water quality and climate regulation through carbon storage (Donato et al., 2011; Lovelock & Reef, 2020; Murdiyarso et al., 2015), pollutant uptake and nutrient cycling (UNEP, 2014), food and nursery grounds for commercially important fishes and invertebrates (Aburto‐Oropeza et al., 2008; Ronnback, 1999), and nesting habitat for coastal birds. Value estimations of mangrove ecosystem services range from US$4217 to $6938 per km^2^ annually (Himes‐Cornell et al., 2018).

Globally, mangrove extent has decreased markedly, but has slowly recovered since 2000 in some areas (FAO, 2007; Giri et al., 2015). Global mangrove extent declined 0.66−1% annually in the 1980s, 3−5 times higher than the rate of global forest loss. An estimated one third of natural mangrove extent was lost from 1950 to 2000 (Alongi, 2002; FAO, 2007) due to timber production and conversion to aquaculture ponds and farmlands (Alongi, 2002; Bryan‐Brown et al., 2020; Murdiyarso et al., 2015). The rate of loss slowed to an average of 0.13% annually from 2000 to 2016 (Goldberg et al., 2020) due to natural recovery, active restoration (at sites where mangroves had previously occurred), and afforestation (at sites where mangroves had not previously occurred). However, recent estimates suggest that 69% of the mangrove area in Indo‐Pacific islands (Lovelock et al., 2015) and half of mangrove coverage worldwide (Alongi, 2015) are still threatened by sea‐level rise, sediment subsidence, and low sediment accretion rate, although there is considerable uncertainty in these estimates (Schuerch et al., 2018).

**FIGURE 1:**
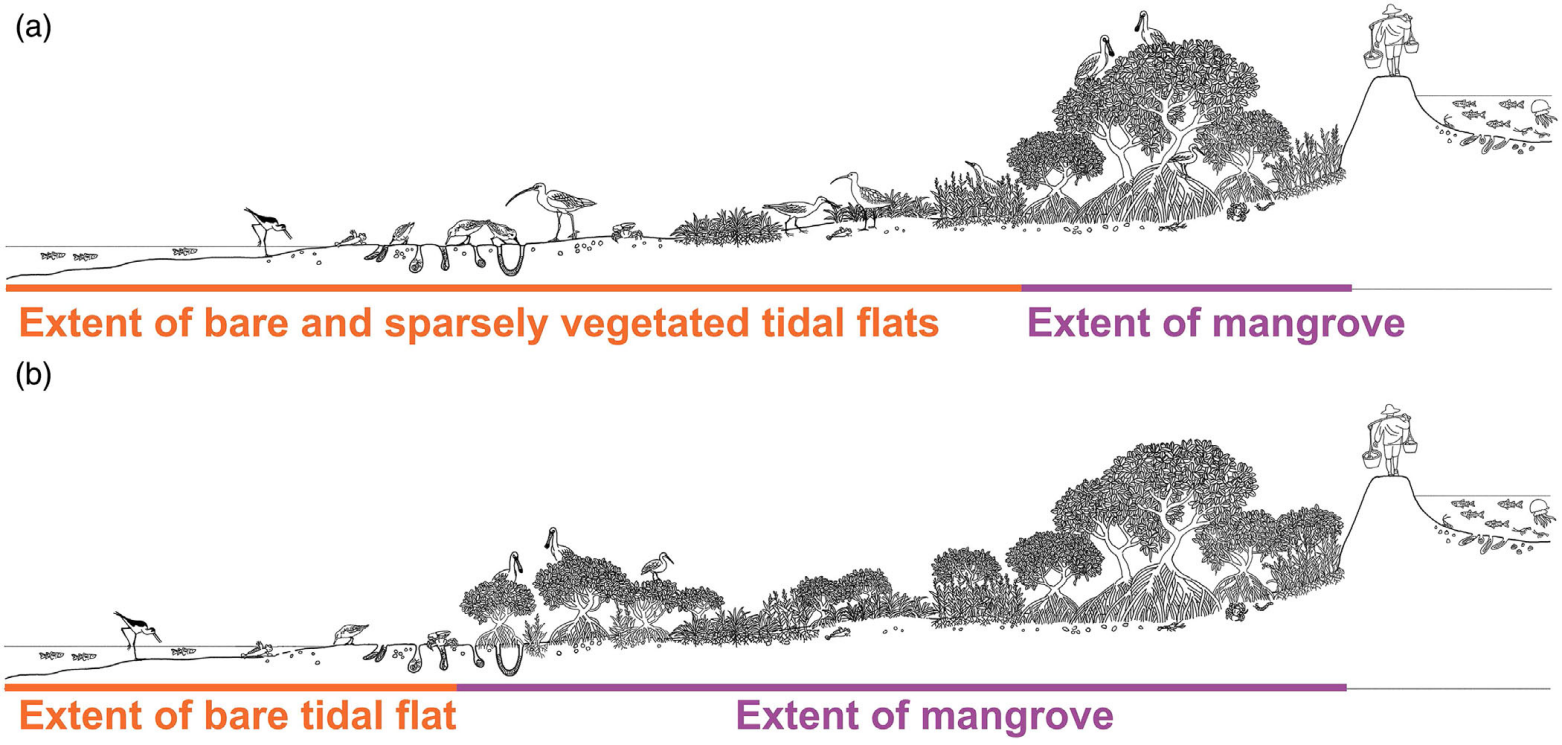
Effects of mangrove expansion on saltmarsh and tidal flats: (a) before mangrove expansion and (b) after mangrove expansion, based on the typical coastal landscape of the Leizhou Peninsula, China, at mid‐ to high tide

Although the boundaries between mangrove, saltmarsh, and open tidal flat ecosystems are highly dynamic and vary between years due to factors such as changes in sedimentation rate, salinity, and accessibility by propagules (Cavanaugh et al., 2019; Saintilan et al., 2014), analysis of satellite images combined with ground truthing indicates rapid expansion of mangroves in many places around the world. These increases are due to natural dynamics (when the rate of sediment accretion exceeds the combined rate of subsidence and sea‐level rise [Lovelock et al., 2015; Schuerch et al., 2018]) and active restoration and afforestation (Jia et al., 2018). Near their latitudinal limits, mangroves have expanded into space originally occupied by saltmarsh, a phenomenon observed on at least 5 continents, including the east coast of Australia (Giri & Long, 2016; Saintilan et al., 2014). Natural expansion has also occurred in New Zealand and Hong Kong, but this is primarily expansion from the upper tidal flat toward the lower tidal flat (Chan et al., 2011; Stokes et al., 2010). In addition to natural expansion, mangroves in several countries, such as China, Bangladesh, Cuba, and Indonesia, have recently increased through active planting (Chen et al., 2009; FAO, 2007; Lee et al., 2019; Spalding et al., 1997). In China (Figure 1), the extent of mangroves increased by 20% (from 18,702 to 22,419 ha) from 2000 to 2015 following the introduction of policies to protect and plant mangroves to safeguard key ecosystem services, such as carbon sequestration and storage, shoreline protection, and food provision (Jia et al., 2018). Indeed, efforts to restore ecosystem services and enhance biodiversity in mangrove forests are increasing globally (Bayraktarov et al., 2020).

### The challenge of conserving mangroves, tidal flats, and shorebirds

During the nonbreeding season, coastal shorebirds primarily forage at low tide when tidal flats are most exposed and rest during high tide when tidal flats are largely inundated (Choi, 2015; Choi et al., 2014; Choi et al., 2019). Shorebirds usually forage on exposed tidal flats and they rarely use densely vegetated areas such as mangroves and saltmarsh, although a small number of shorebird species roost in mangroves (Choi et al., 2017; Zwarts, 1988).

Recent increases in mangrove distribution have led to a conservation dilemma in some areas where mangrove expansion has replaced saltmarsh and bare tidal habitats—in many cases, critical shorebird habitat (Figure 1 & Appendix S1; Huang et al., 2012; Straw & Saintilan, 2006; Zou et al., 2006). This dilemma is particularly pertinent along the East Asian‐Australasian Flyway (EAAF), which encompasses 45.6% of the global mangrove extent (Giri et al., 2011) and has the highest proportion (23%, 46 species) of threatened migratory waterbirds (Kirby et al., 2008). As one of the prominent groups among waterbirds, many migratory shorebird populations along the coast of EAAF have declined substantially, and this is linked to extensive loss of tidal flats along their refueling sites (Piersma et al., 2016; Studds et al., 2017); 11 shorebird species are currently listed as threatened under International Union for the Conservation of Nature categories vulnerable, endangered, or critically endangered, and an additional 10 species are listed as near threatened (IUCN, 2021).

Many countries have regulations directed at mangrove conservation or restoration, and some also have regulations to protect shorebirds or their tidal flat habitats. There is often a lack of clear guidance on how to resolve the potential conflict caused by this competition between mangroves and the habitats used by declining shorebird populations. We considered a number of cases in which this conflict has emerged and conducted an empirical investigation along the coastline of southern China by way of a worked example, building on earlier work that mapped the extent of mangroves (Jia et al., 2018) and documented important shorebird sites (Jackson et al., 2021). We also devised a decision tree to assist managers in addressing this dilemma and ensuring that conservation of mangroves does not imperil migratory shorebirds.

## METHODS

### Case studies

**FIGURE 2:**
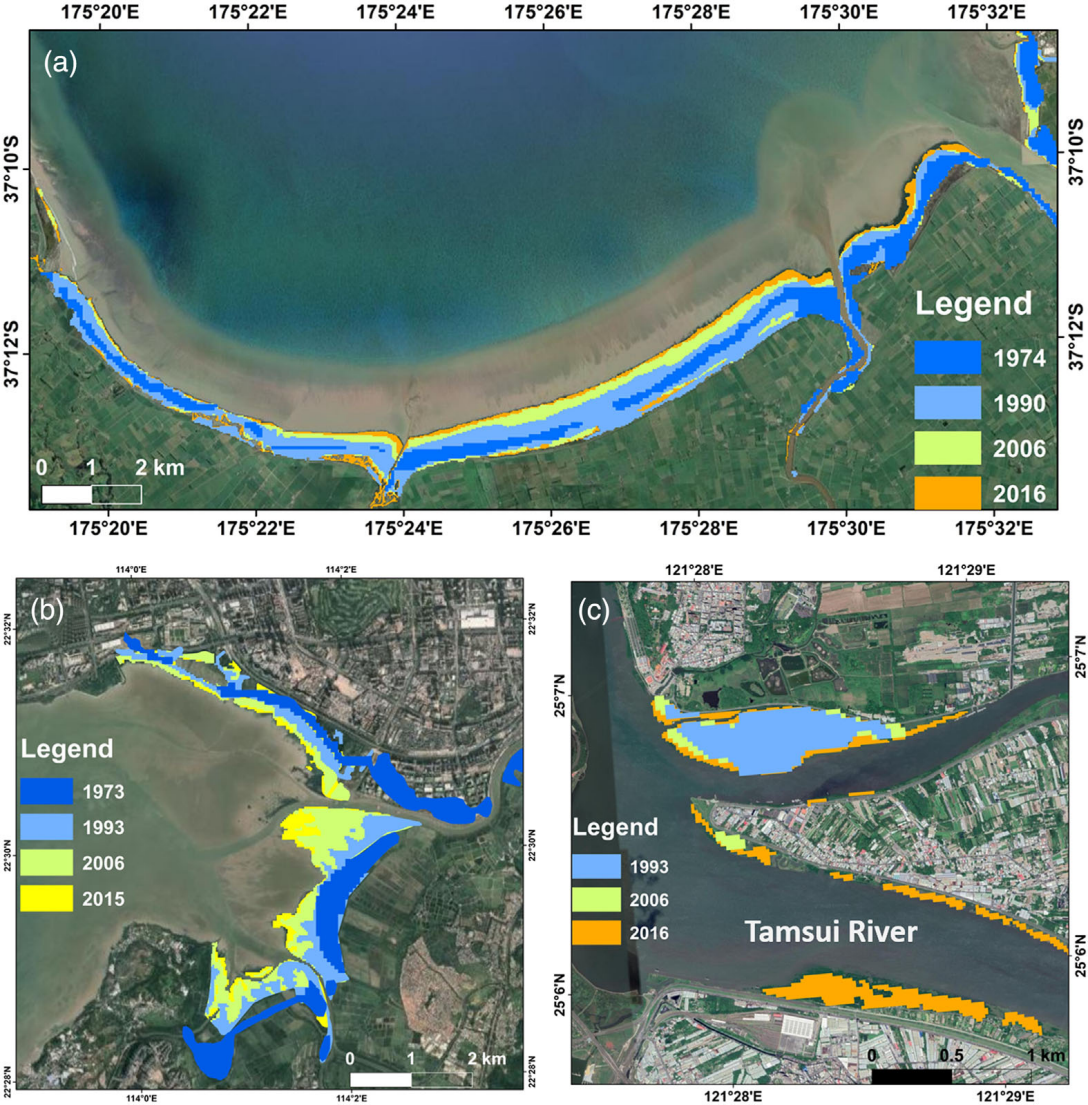
Spread of mangroves in 3 of the 5 case studies: (a) south of the Firth of Thames, North Island of New Zealand, where mangroves covered 6.14 km^2^ in 1974 and 15.01 km^2^ in 2016); (b) Shenzhen Bay, China mainland, where mangrove covered 3.1 km^2^ in 1973 and 5.02 km^2^ in 2015; and (c) Taiwan Island, where mangrove expansion in Guandu and the southern side of Tamsui River was from 0.32 km^2^ in 1993 to 0.65 km^2^ in 2016. Mapping method details can be found in “Quantifying national change in mangrove and tidal flat extent” section. Base image composites were developed with Landsat data, courtesy of Google Earth and Maxar Technologies 2021

Through personal experiences and concerns raised by local conservation practitioners, we became aware of 5 examples across the EAAF in which mangroves have expanded into saltmarshes or tidal flats at internationally important shorebird sites: the Firth of Thames on the North Island of New Zealand; Hunter Valley in Eastern Australia; Leizhou Peninsula and Shenzhen Bay in southern China; and Guandu on Taiwan Island (Table 1). All but 1 of these locations (Guandu) is designated as a wetland of international importance under the Ramsar convention (Ramsar, 2017), and all but 1 (Leizhou Peninsula) is an important bird area (BirdLife International, 2020). Some of these sites provide habitat for highly threatened shorebird species, such as the black stilt (*Himantopus novaezelandiae*) (critically endangered), spoon‐billed sandpiper (*Calidris pygmaea*) (critically endangered), and eastern curlew (*Numenius madagascariensis*) (endangered). All sites are protected to some extent and have had mangrove expansion in recent years through natural dispersal or artificial afforestation (Figure 2) (Jia et al., 2018; Reid, 2019). Shorebird population and distribution changes have occurred in Hunter Valley and Guandu (Appendix S5). At some sites, local managers and stakeholders have physically removed mangrove seedlings (Firth of Thames, Hunter Valley and Shenzhen Bay) or mature mangroves (Hunter Valley) to slow down mangrove expansion and preserve shorebird habitat. However, local and national‐level policies can inhibit mangrove management. For example, Australia has extensive regulations around mangrove removal (Australian Government, 2014), and regulations that prohibit the removal of mangroves have been implemented at Shenzhen Bay (National People's Congress, 2021 [Marine Environmental Protection Law of the People's Republic of China]). Nonetheless, migratory shorebird habitats, including tidal flats, are also protected in Australia and China (Australian Government, 2014; National People's Congress, 2020), leading to potentially conflicting management goals in such areas. Lengthy consultation or lack of awareness has led to inaction in 1 location (Guandu), causing ongoing reduction in foraging and roosting habitat for threatened shorebirds. Competition for space is particularly severe at Leizhou, which comprises nearly one‐third of the total mangrove area in mainland China. This site also holds the largest spoon‐billed sandpiper wintering population in the country (Spoon‐billed Sandpiper Conservation Alliance, 2020). Detailed descriptions of each of these 5 case studies are given in Appendix S5.

**TABLE 1:** Summary of the mangrove‐shorebird interactions in the 5 case studies

Site name	Number of shorebirds supported	Local conditions	Management measures	Consequences	Site importance
Firth of Thames, New Zealand	35,000	native mangroves expanded to bare tidal flats	seedling removal	slowed the speed of mangrove expansion	IBA, Ramsar
Hunter Valley, Australia	∼5000–10000	mangrove expansion	initial mature mangrove removal; ongoing seedling removal	mangroves removed from shorebird habitat; roosting habitat increased; sharp‐tailed sandpipers increased	IBA, Ramsar
Shenzhen Bay, mainland China	c40,000	mangrove expanded to bare tidal flats	seedling removal	slowed the speed of mangrove expansion	IBA, Ramsar
Guandu, Taiwan	3000	mangrove expanded to bare tidal flats and saltmarsh areas	no intervention	shorebirds displaced	IBA
Leizhou Peninsula, mainland China	∼25,000	mangrove restored and afforested on bare tidal flats	active plantation	potential negative impact on shorebirds	Ramsar

Abbreviation: IBA, important bird area.

### Quantifying national change in mangrove and tidal flat extent

To gain insight into whether our 5 examples are isolated cases or an emerging broad‐scale problem, we quantified change in mangrove extent from 2000 to 2015 and change in tidal flat extent in important coastal shorebird sites in mainland China. We used the methods described in Jia et al. (2018) for mangroves and Jackson et al. (2021) for shorebird sites. As described in Jackson et al. (2021), the list of important coastal shorebird sites in mainland China was derived from Bai et al. (2015) and Conklin et al. (2014), which report coastal sites of international importance for shorebird species (i.e., sites meet Ramsar Convention listing criterion 6, hosting >1% of the flyway population). We considered only shorebird sites in Zhejiang or farther south because only these overlap the distribution of mangroves. The coastline of all sites comprises a hardened seawall, which is generally straightforward to discern and interpret in images or image composites in Google Earth. We therefore used the historical image tool in Google Earth to manually map the 2015 coastline of each site with the Google Earth path tool based on site boundaries of national nature reserves (National Earth System Science Data Center – National Science & Technology Infrastructure of China, 2016); survey routes of sites identified in Bai et al. (2015) obtained from local observers involved in the China Coastal Waterbird Census; or a 3‐km stretch of coastline on either side of the coordinates for additional sites from Conklin et al. (2014) not included in site boundaries or survey routes. The individual image sources and dates viewed in Google Earth to map the coastline for each site are in Appendix S3.

To map change in mangrove extent, we followed methods described in Jia et al. (2018). We adopted an object‐based classification approach to interpret mangrove forests from Landsat images and used eCognition Developer 9.2 to process the classification. We obtained homogeneous objects by segmenting images based on 3 parameters: scale, compactness, and shape (Jia et al., 2018). Then we processed the nearest neighbor classifier (NN classifier) to classify objects into specific categories (mangrove or nonmangrove). We postprocessed the classification output by manually correcting misclassified objects (89−94% overall accuracy [Jia et al., 2018]). The difference in mangrove extent in 2000 and 2015 for each of the important coastal shorebird sites was then calculated (square kilometers).

To estimate tidal flat change, we followed the methods of Jackson et al. (2021). The extent of tidal flats at each important shorebird site in 2000 and 2015 was estimated using data from global tidal flat maps from 1999 to 2001 and 2014 to 2016 (https://intertidal.app/ [Murray et al., 2019]). Following the Jackson et al. (2021) approach, an “area of interest” for each important shorebird site was developed and extended from the coastline of the site (mapped manually for each year with Google Earth, see above) to the seaward extent of tidal flats parallel to the coastline. For each site, the 1999–2001 tidal flat map layer and the 2014–2016 tidal flat map layer were clipped to the area of interest, and then the area of tidal flats in 1999–2001, the area of tidal flats in 2014–2016, and the percent change between the 2 were calculated. Data for site boundaries and mangrove extent are available from https://osf.io/d76tq/.

The areal extent of mangrove and tidal flat in 2000 and 2015 was compared using nonparametric paired 2‐sample Wilcoxon test (because data were not normally distributed) in R 3.6.3 (R Core Team, 2020). We used *p* < 0.05 as the significance level.

### Decision tree approach

To help inform local decision‐making on how and when to implement management actions on mangroves, we designed a decision tree that provides step‐by‐step guidance (Figure 4). In the first step, decision makers examine the area of interest and determine its importance to shorebirds (Figure 4, Q1). Some shorebirds have multiple foraging and resting sites and might switch if their original site is degraded or lost, whereas others do not (Zhang et al., 2018). This redundancy might lead to certain sites being considered nonessential for shorebird conservation (Dhanjal‐Adams et al., 2017). To address this problem, one could determine the presence of threatened shorebird species with the IUCN Red List of Threated Species or occurrence records on shorebird distribution patterns. If the area is of low importance to shorebirds (abundances or species threat status does not meet Ramsar site criteria [Ramsar, 2017]), then there is not a strong trade‐off between mangrove restoration and afforestation and shorebird conservation. The decision makers might move on to considering other factors involved, such as economic cost and carbon sequestration capacity, in the decision to manage mangroves.

In the second step, for sites that were identified in step one as being important to shorebirds, decision makers estimate the potential impacts of mangrove expansion (Figure 4, Q2). Remote sensing and field survey data may be used to quantify trends in mangrove and tidal flat extent (Jia et al., 2021; Swales et al., 2009), and plantation plans (spatial extent and quantity) by local stakeholders should also be considered (Choi et al., 2017; Sievers et al., 2020). For example, mangrove restoration through converting some aquaculture ponds back to mangroves is likely to have a lower impact on shorebird habitats than mangrove afforestation on open tidal flats where mangroves did not previously occur. It is also important to ensure that sufficient high‐tide roosting habitat (which can include aquaculture ponds; Jackson et al., 2020) is retained. Even where a low impact of mangrove expansion is expected, we suggest a conservative approach of monitoring before and during the implementation of afforestation (McDonald‐Madden, Probert, et al., 2010).

In the third step, where mangrove expansion is likely to result in loss of important shorebird habitat, decision makers could estimate the value of mangrove ecosystems to objectively assess the shorebird–mangrove trade‐off (Figure 4, Q3). Several tools are available to evaluate the value of an ecosystem, such as the System of Environmental Ecosystem Accounts (United Nations, 2021), which was published to create a set of ecosystem accounts (extent account, condition account, and ecosystem services account), in both physical and monetary values, to record the value of and changes in ecosystem and ecosystem services (Sanchirico et al., 2015; UN Sustainable Development, 2017). After the assessment, if the mangrove ecosystem value is considered lower than the original ecosystem value with shorebirds and other habitat features (e.g., tidal flat, saltmarsh), the implication is clear—managers would limit the further spread of mangroves. For example, if local policy sets very high values on endangered shorebirds, this might mean compulsory protection of particular species regardless of the ecological value of alternative policies (Lau & Shi, 2000). Besides, planting mangroves in the wrong place could have negative impacts on other components of the coastal ecosystem, such as saltmarsh and sea grass, because those vulnerable ecosystems could be trampled by planting volunteers or overgrown by mangroves, a problem that could worsen if non‐native mangrove species are planted (Wetlands International, 2016).

If the decision is finely balanced, a conflict resolution process is entered into that seeks to investigate carefully the trade‐offs between planting mangroves for high‐value service provision and preserving original habitats for endangered shorebirds and other habitat types (Xiao et al., 2018). In this case, more complex frameworks are needed, such as structured decision‐making or working through a mitigation hierarchy as part of an environmental impact assessment (Jacob et al., 2016; McDonald‐Madden, Baxter, et al., 2010). We used 1 of our 5 case studies—Shenzhen Bay—to illustrate implementation of the decision tree for conservation decision‐making.

## RESULTS

### Mangrove and shorebird dilemma in China

**FIGURE 3:**
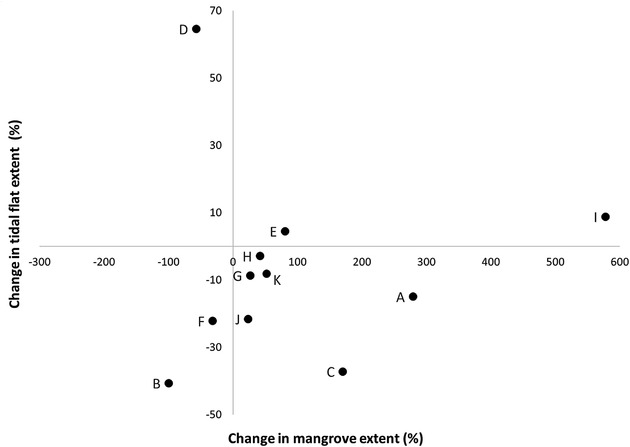
Change in extent of mangrove and tidal flat at 14 important shorebird sites in southern China (A, Quanzhou Bay; B, Haicang Coast; C, Liuhewei; D, Haifeng Nature Reserve; E, Futian National Nature Reserve [NNR]; F, Xitou coast; G, Zhanjiang NNR; H, Beihai coast; I, Yujiang Village; J, Guangxi Beilun Estuary NNR; K, Sigeng Provincial Nature Reserve). Four sites are excluded: 1 site where data on the extent of tidal flats were not available and 3 sites where mangroves were absent in 2000 but present in 2015 (all of these 3 sites had decreasing tidal flat extent)

Within the 22 important coastal shorebird sites we mapped in southern China, mangroves were present in 14 sites. At 11 sites mangrove extent increased, and in 3 mangrove extent decreased from 2000 to 2015. The areal extent of mangrove in 2015 (all sites combined total = 58 km^2^) was significantly larger than that in 2000 (all sites combined total = 42.2 km^2^) (Wilcoxon signed rank test, *V* = 8, *p* < 0.01, *n* = 14). Among the 11 sites with an increase in mangrove extent, expansion was 16 km^2^ (max = 8.5 km^2^, min = 0.1 km^2^, *n* = 11), at a mean rate of 0.1 km^2^ (SE = 0.05) per site per year (*n* = 11) and an overall mean change of 156.4% (SE = 67.7) (*n* = 8 with 3 other sites excluded in the calculation of mean because no mangroves occurred in 2000). In contrast, the areal extent of bare tidal flat in 2015 was significantly smaller than in 2000 (Wilcoxon signed rank test, *V* = 225, *p* < 0.01, *n* = 22). Among the 11 sites with mangrove expansion, the extent of bare tidal area declined in 9 sites (81.8%) (max = 23.7 km^2^, min = 0.13 km^2^, *n* = 9) at a mean rate of −0.43 km^2^ (0.16) per year (*n* = 9) and an overall decrease of 18.3% (4.1) (*n* = 9) (Figure 3 & Appendix S4).

### Decision tree application

**FIGURE 4:**
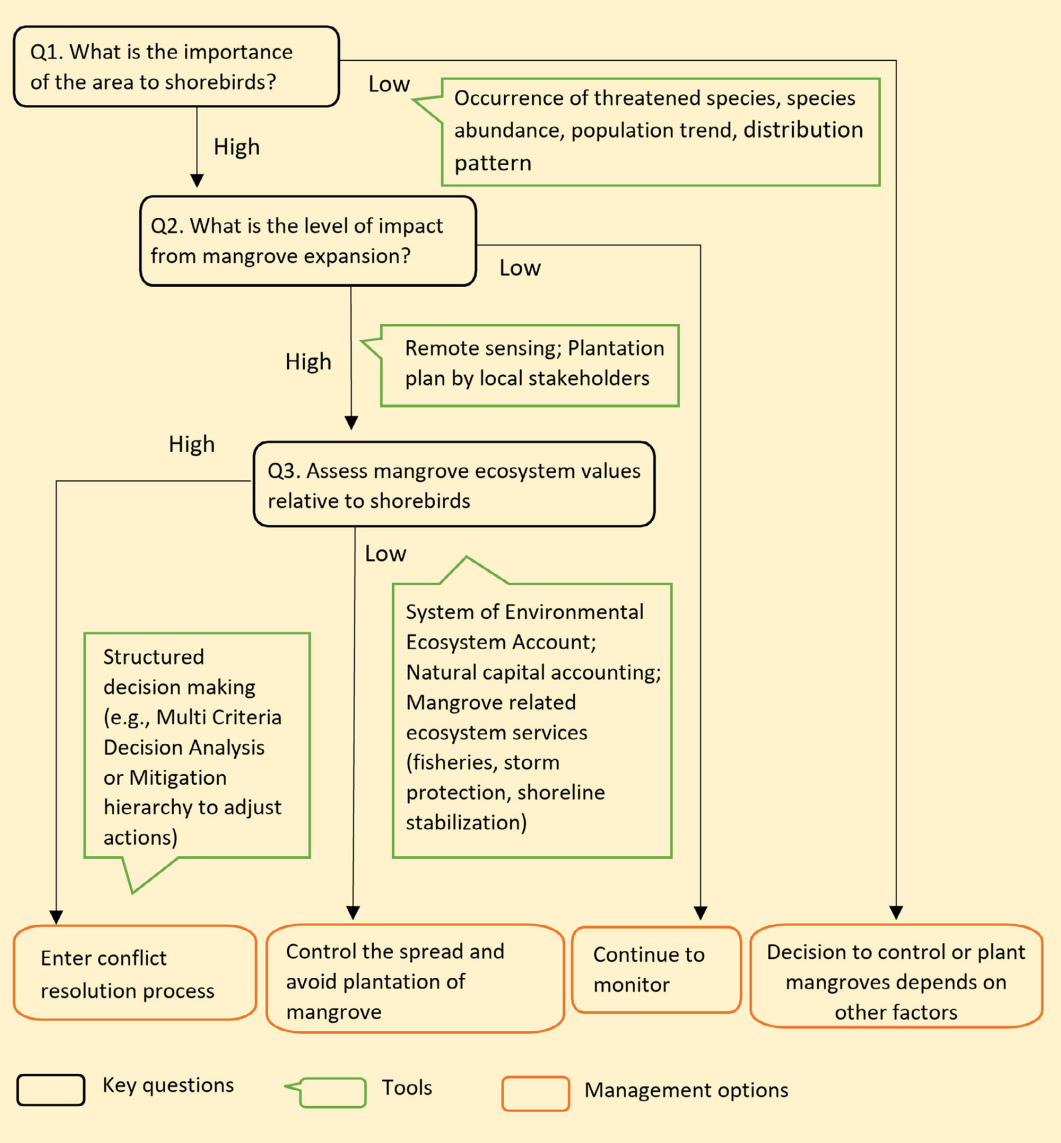
Decision tree for determining management options to restore mangroves or conserve tidal flats for shorebirds

Shenzhen Bay is located at the Pearl River Estuary in southeast China. The wetlands around the bay provide important wintering and stopover habitats for about 40,000 migratory shorebirds (Choi et al., 2020; Sung et al., 2021), including 4 critically endangered or endangered and 9 near‐threatened shorebird species (Figure 4, Q1‐High). Mangroves have expanded in the bay for the last 3 decades, mainly through natural dispersal (Figure 2b). This could significantly affect shorebird populations, many of which are declining significantly (Choi et al., 2020; Studds et al., 2017), by reducing the extent of their foraging areas. As a result, local managers and stakeholders have actively removed mangrove seedlings or cut the stumps of mangrove trees to slow down mangrove expansion and preserve open tidal flats for shorebird to forage (Agriculture Fisheries & Conservation Department, 2011) (Figure 4, Q2‐High). However, those efforts were not always supported by local policies due to the conflicting demands of mangroves and shorebirds (Agriculture Fisheries & Conservation Department, 2011), making it prudent to implement research (e.g., natural capital accounting) to assess the mangrove ecosystem values relative to shorebirds (Figure 4, Q3). Once this has been achieved, managers might return to the decision tree to decide whether avoidance of further mangrove spread is warranted.

## DISCUSSION

Our results demonstrate the importance of taking a holistic view of coastal ecosystem management. Considering any particular component, such as mangroves or tidal flats, separately from the others could lead to unintended negative consequences for the whole system. Despite an overall global decline in their extent, mangroves have expanded in many areas of the world due to active restoration, afforestation, and natural dispersal. However, our results demonstrate that this expansion can lead to a dilemma between mangrove and shorebird management in conservation decision‐making. This problem has especially come to the fore in regions where migratory shorebird populations are declining rapidly. Our decision tree can help guide decision‐making in situations of potential conflict. It can be used to formulate optimal solutions and ensure that mangrove restoration or afforestation does not lead to negative impacts on other important ecosystem components, particularly the increasingly threatened shorebirds that depend on these coastal habitats. In many cases, a regional approach may be needed to ensure that effective mangrove conservation is achieved alongside meeting other coastal conservation goals.

Among the important shorebird sites with mangroves present in mainland China, a majority (79%) showed mangrove expansion from 2000 to 2015, suggesting that the dilemma between mangrove and shorebird conservation has become widespread in southern China. In mainland China, there are policies such as the Forest Law of the People's Republic of China and the Wetland Protection Law that provide guidelines on mangrove protection and management. China's unprecedented investment in sustainability has led to the implementation of a series of ecological projects to restore natural habitats (Bryan et al., 2018), such as the National Coastal Shelterbelt System Construction Project Plan (2016–2025), which together with other similar projects has resulted in a marked increase in the areal extent of mangrove forest since 2000, mainly through afforestation and restoration (Jia et al., 2018; Paulson Institute, 2020). The latest mangrove conservation and restoration action plan aims to increase the current extent of mangrove forest by at least 30% by 2025 through active planting and substantial financial support to local departments (The Ministry of Natural Resources & the National Forestry & Grassland Administration, 2020; The State Council of the People's Republic of China, 2020). China has also seen a significant conservation effort directed toward migratory shorebirds and their habitats in recent years (Choi et al., 2022; Yang et al., 2020), for example, through the inscription of 3 tidal sites onto the World Heritage list in 2019 and another ∼14 sites to be considered for World Heritage Listing within the next 3 years, with nominations based in large part on the importance for threatened waterbirds (IUCN, 2019).

Such a rapid and positive change in attitude toward nature conservation is encouraging, but also poses a challenge to ecosystem managers (Fan et al., 2020)—in this case ensuring the costs and benefits of mangrove restoration or afforestation effort are fully evaluated so that potential negative impacts on shorebirds or other coastal conservation values can be carefully mitigated. Good decision‐making will balance the relative benefits of mangrove expansion and tidal flat conservation by taking into account which areas are of highest importance to biodiversity, including shorebirds.

## Supporting information

Figure S1: Spoon‐billed Sandpipers *Calidris pygmaea* – a Critically Endangered shorebird species, were foraging on tidal flat with new mangrove shoots planted nearby at the Zhanjiang Mangrove National Nature Reserve, China.
Figure S2: Satellite images (see the end of this document) showing the spread of mangroves in the 14 sites (Wenzhou, Minjiang, Meizhou, Quanzhou, Haicang, Liuhewei, Haifeng, Futian (only Futian NNR's area was quantified for table S1), Xitou, Zhanjiang NNR (only a small part was shown due to the large size of the NNR), Beihai, Yujiang, Beilun Estuary NNR and Sigeng) where mangroves were present in the national analysis, with blue denotes the extent of mangrove in 2000 while orange for 2015. Base image composites were developed with Landsat data, courtesy of Google Earth and Maxar Technologies 2021.
Table S3: The individual image source(s) and date(s) that were viewed in Google Earth to map the coastline for the 22 important shorebird sites in mainland China, together with the key shorebird species that these sites support. Reference source 1 denotes Bai et al. (2015), 2 Conklin et al. (2014), 3 Spoon‐billed Sandpiper Conservation Alliance (2020) and 4 Choi et al. (2020).
Table S4: The extent and change in mangroves and tidal flats among the 22 important shorebird sites in mainland China between 2000 and 2015.
