# Supplementary material for: An emerging coastal wetland management dilemma between mangrove expansion and shorebird conservation
Source: Conserv Biol. 2022 Jun 8;36(5):e13905. doi: 10.1111/cobi.13905 (PMC9912193; doi:10.1111/cobi.13905)
Supplement: Supplementary file 1 — Figure S1: Spoon‐billed Sandpipers Calidris pygmaea – a Critically Endangered shorebird species, were foraging on tidal flat with new mangrove shoots planted nearby at the Zhanjiang Mangrove National Nature Reserve, China. Figure S2: Satellite images (see the end of this document) showing the spread of mangroves in the 14 sites (Wenzhou, Minjiang, Meizhou, Quanzhou, Haicang, Liuhewei, Haifeng, Futian (only Futian NNR's area was quantified for table S1), Xitou, Zhanjiang NNR (only a small part was shown due to the large size of the NNR), Beihai, Yujiang, Beilun Estuary NNR and Sigeng) where mangroves were present in the national analysis, with blue denotes the extent of mangrove in 2000 while orange for 2015. Base image composites were developed with Landsat data, courtesy of Google Earth and Maxar Technologies 2021. Table S3: The individual image source(s) and date(s) that were viewed in Google Earth to map the coastline for the 22 important shorebird sites in mainland China, together with the key shorebird species that these sites support. Reference source 1 denotes Bai et al. (2015), 2 Conklin et al. (2014), 3 Spoon‐billed Sandpiper Conservation Alliance (2020) and 4 Choi et al. (2020). Table S4: The extent and change in mangroves and tidal flats among the 22 important shorebird sites in mainland China between 2000 and 2015. [file COBI-36-0-s001.pdf]

Supplementary materials for

An emerging coastal wetland management dilemma between mangrove expansion and shorebird conservation

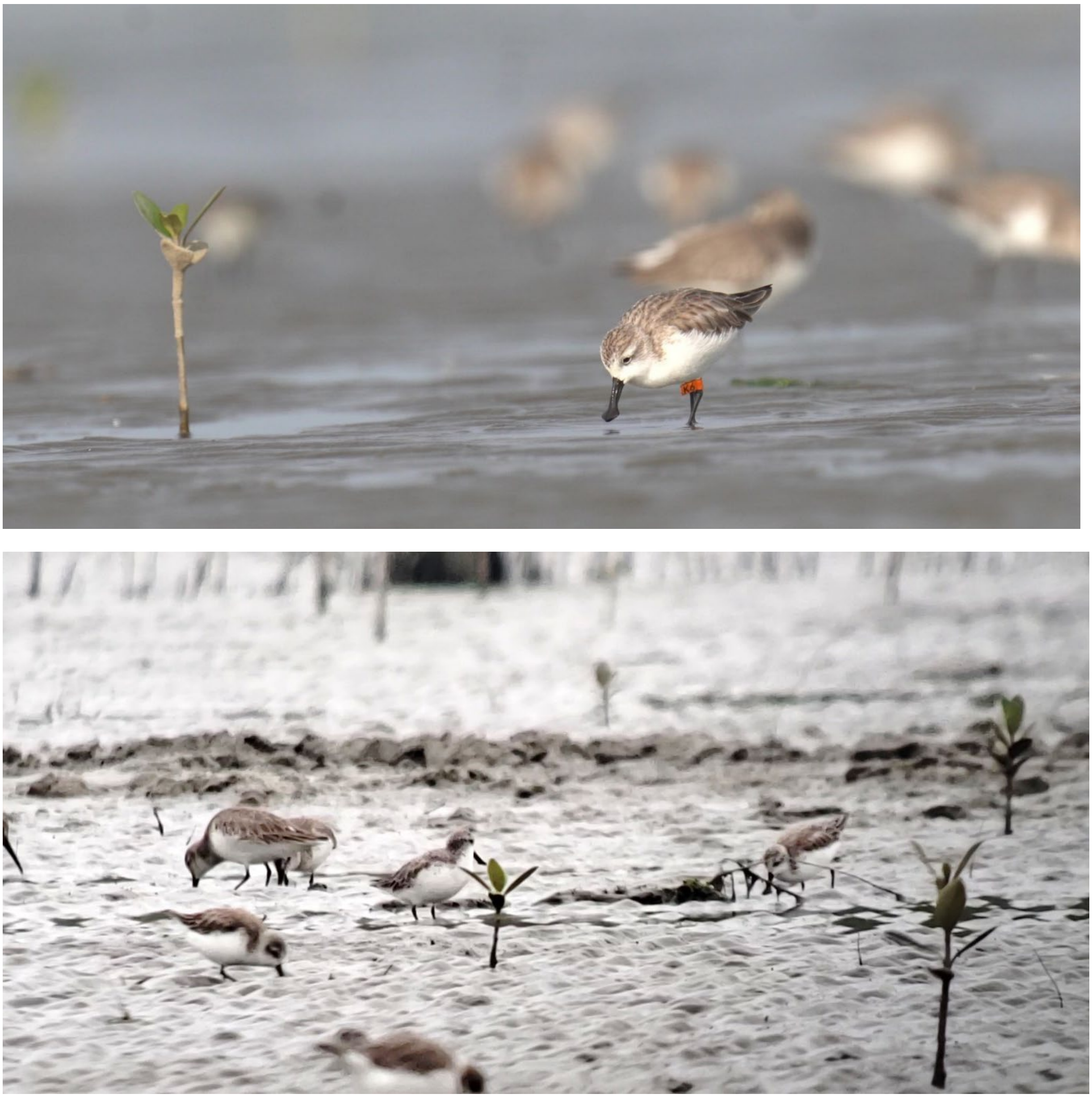

Figure S1: Spoon-billed Sandpipers *Calidris pygmaea* – a Critically Endangered shorebird species, were foraging on tidal flat with new mangrove shoots planted nearby at the Zhanjiang Mangrove National Nature Reserve, China.

Figure S2 (see the end of this document): Satellite images showing the spread of mangroves in the 14 sites (Wenzhou, Minjiang, Meizhou, Quanzhou, Haicang, Liuhewei, Haifeng, Futian (only Futian NNR's area was quantified for table S1), Xitou, Zhanjiang NNR (only a small part was shown due to the large size of the NNR), Beihai, Yujiang, Beilun Estuary NNR and Sigeng) where mangroves were present in the national analysis, with blue denotes the extent of mangrove in 2000 while orange for 2015. Base image composites were developed with Landsat data, courtesy of Google Earth and Maxar Technologies 2021.

Table S3: The individual image source(s) and date(s) that were viewed in Google Earth to map the coastline for the 22 important shorebird sites in mainland China, together with the key shorebird species that these sites support. Reference source 1 denotes Bai et al. (2015), 2 Conklin et al. (2014), 3 Spoon-billed Sandpiper Conservation Alliance (2020) and 4 Choi et al. (2020).

Site	Image source(s) for 2015 coastline mapping (accessed through Google Earth Historical Imagery tool)	Image date(s)	Shorebird species with >1% of the flyway population (reference source)
Hangzhou Wan	Maxar Technologies; CNES / Airbus	19-Feb-2015	Spoon-billed Sandpiper (2), Bar-tailed Godwit, Black-tailed Godwit, Common Greenshank, Common Redshank, Dunlin, Grey Plover, Greater Sand Plover, Lesser Sand Plover, Kentish Plover, Long-billed Plover (4)
Sanmen Wan	CNES / Airbus	12-Mar-2015	Ruddy Turnstone (2)
Yueqing Wan	Maxar Technologies	8-Sep-2015	Dunlin (2)
Wenzhou Wan & Xuanmen Wan	Maxar Technologies	16-Jun-2015	Dunlin, Spoon-billed Sandpiper (2), Black-tailed Godwit, Broad-billed Sandpiper, Eurasian Curlew, Grey Plover, Kentish Plover, Long-toed Stint, Lesser Sand Plover, Nordmann's Greenshank, Pied Avocet, Red Knot, Sharp-tailed Sandpiper (4)
Minjiang Estuary National Nature Reserve	Maxar Technologies	11-Apr-2014	Kentish Plover, Lesser Sand Plover, Greater Sand Plover, Terek Sandpiper, Ruddy Turnstone, Sanderling, Spoon-billed Sandpiper, Spotted Redshank (1,4)
Xinghua Wan	Maxar Technologies	18-Jan-2015	Spoon-billed Sandpiper, Dunlin (2)
Meizhou Wan	CNES / Airbus; Landsat/Copernicus; Maxar Technologies	13-Apr-2015; 14-Apr-2015	Dunlin (2)
Quanzhou Bay	CNES / Airbus; Landsat/Copernicus; Maxar Technologies	31-Dec-2014; 15-Jun-2013	Kentish Plover, Grey Plover, Greater Sand Plover, Lesser Sand Plover, Whimbrel,

			Eurasian Curlew, Pied Avocet, Dunlin, Curlew Sandpiper, Terek Sandpiper, Grey-tailed Tattler, Sanderling (1,4)
Dadeng Island & Weitou Bay	CNES / Airbus; Landsat/Copernicus; Maxar Technologies	4-Oct-2014; 16-Oct-2014	Greater Sand Plover, Whimbrel, Nordmann's Greenshank, Spoon-billed Sandpiper, Broad-billed Sandpiper (1)
Xiamen Coast (incl. Aotou & Fenglin)	Maxar Technologies	1-Aug-2015	Spoon-billed Sandpiper (2)
Haicang Coast, Xiamen	Maxar Technologies	16-Oct-2014; 30-Dec-2104	Spoon-billed Sandpiper (2)
Liuhewei, Shantou	Maxar Technologies	13-May-2015	Spoon-billed Sandpiper (2)
Nangankou, Shantou	Maxar Technologies	13-May-2015	Spoon-billed Sandpiper (2)
Haifeng Wetlands Nature Reserve	CNES / Airbus; Landsat/Copernicus; Maxar Technologies	06-Sep-2014; 18-Jan-2015; 17-Oct-2015; 23-Aug-2015;	Spotted Redshank, Wood Sandpiper (1, Choi *pers.obs*.)
Futian Mangrove National Nature Reserve	Maxar Technologies	14-Apr-2015	Pied Avocet, Kentish Plover, Black-tailed Godwit, Spotted Redshank, Common Redshank, Common Greenshank, Curlew Sandpiper (1)
Nansha Wetland	Maxar Technologies	21-Oct-2015	Pied Avocet (1)
Xitou coast	CNES / Airbus	9-Oct-2014	Lesser Sand Plover, Spoon-billed Sandpiper (1)
Zhanjiang Mangrove National Nature Reserve	CNES / Airbus; Landsat/Copernicus	14-Jun-2014; 16-Nov-2014; 18-Dec-2015; 16-Sep-2016	Spoon-billed Sandpiper (3), Kentish Plover (4)
Beihai coast	Maxar Technologies	13-Apr-2015	Greater Sand Plover (1), Lesser Sand Plover, Sanderling (4)
Yujiang Village, Xiangli Town	CNES / Airbus	17-Dec-2015	Spoon-billed Sandpiper (2)
Guangxi Beilun Estuary National Nature Reserve	CNES / Airbus; Maxar	06-Oct-2015; 18-Oct-2015; 20-Oct-2015;	Spoon-billed Sandpiper (2)

Sigeng Provincial Nature Reserve	CNES / Airbus; Landsat/Copernicus	5-Sep-2015	Lesser Sand Plover (2)

Table S4: The extent and change in mangroves and tidal flats among the 22 important shorebird sites in mainland China between 2000 and 2015.

Site	Province	Mangrove area (km^2)			Tidal flat (km^2)		
		**Year 2000**	**Year 2015**	**% change**	**Year 2000**	**Year 2015**	**% change**
Hangzhou Wan	Zhejiang	0.0	0.0	Mangrove absent	17.2	2.8	-83.7
Sanmen Wan	Zhejiang	0.0	0.0	Mangrove absent	14.3	8.7	-39.2
Yueqing Wan	Zhejiang	0.0	0.0	Mangrove absent	9.9	6.4	-35.4
Wenzhou Wan & Xuanmen Wan	Zhejiang	0.0	0.1	Increased from zero	20.9	13.6	-34.9
Minjiang Estuary National Nature Reserve	Fujian	0.0	1.6	Increased from zero	24.3	17.8	-26.7
Xinghua Wan	Fujian	0.0	0.0	Mangrove absent	16.2	15.2	-6.2
Meizhou Wan	Fujian	0.0	0.1	Increased from zero	32.9	29.7	-9.7
Quanzhou Bay	Fujian	0.4	1.5	279	38	32.3	-15.0
Dadeng Island & Weitou Bay	Fujian	0.0	0.0	Mangrove absent	63	62.1	-1.4
Xiamen Coast (incl. Aotou & Fenglin)	Fujian	0.0	0.0	Mangrove absent	0.73	0.39	-46.6
Haicang Coast, Xiamen	Fujian	0.001	0.0	-100.0	31.7	18.8	-40.7
Liuhewei, Shantou	Guangdong	0.2	0.6	170.1	4.3	2.7	-37.2

Nangankou, Shantou	Guangdong	0.0	0.0	Mangrove absent	0.15	0	-100.0
Haifeng Wetlands Nature Reserve	Guangdong	0.02	0.01	-57.1	3.1	5.1	64.5
Futian Mangrove National Nature Reserve	Guangdong	0.5	0.9	80.6	11.2	11.7	4.5
Nansha Wetland	Guangdong	0.0	0.0	Mangrove absent	0.18	0.22	22.2
Xitou coast	Guangdong	0.5	0.3	-31.9	7.2	5.6	-22.2
Zhanjiang Mangrove National Nature Reserve	Guangdong	31.9	40.5	26.7	272.1	248.4	-8.7
Beihai coast	Guangxi	0.9	1.2	41.9	10.2	9.9	-2.9
Yujiang Village, Xiangli Town	Guangxi	0.3	1.8	577.6	14.8	16.1	8.8
Guangxi Beilun Estuary National Nature Reserve	Guangxi	7.2	8.9	23.4	44.4	34.8	-21.6
Sigeng Provincial Nature Reserve	Hainan	0.3	0.5	51.8	1.61	1.48	-8.1

Appendix S5 - Case studies

Firth of Thames, New Zealand

Firth of Thames (Tikapa Moana), also known as Miranda (37.18°S, 175.35°E), in New Zealand is one of the most important non-breeding sites for shorebirds in the EAAF, supporting more than 35,000 shorebirds annually and was listed as an Important Bird Area and Ramsar site, wetlands of international significance (Bamford et al. 2008; Ramsar 2014; Forest & Bird 2016). The intertidal wetlands at Miranda are important to shorebirds such as the Bar-tailed Godwit *Limosa lapponica* (NT), Red Knot *Calidris canutus* (NT), Wrybill *Anarhynchus frontalis* (VU) and Black Stilt *Himantopus novaezelandiae* (CR). The native Grey mangrove *Avicennia marina* in Miranda has grown rapidly in the southern Firth, almost tripled its area cover from 4 km^2 in the 1950s to 11 km^2 in 2006 (Swales et al. 2007)(Figure 2a). Its expansion came largely at the expense of bare intertidal flat, which is the main foraging and roosting habitats for thousands of shorebird inhabitants. Volunteers have been actively removing mangrove seedlings to slow down the spread and preserve habitats for shorebirds, but their effort was only permitted officially in recent years due to the strict legislation to protect mangroves in the past (Riegen *pers. comm.*).

Hunter Valley, Australia

The Hunter Estuary is an important region for shorebirds in southeast Australia, but most of its wetlands have been significantly modified from their original condition. The region requires significant management effort to control mangroves to maintain habitat values for shorebirds. For example, sections of Ash Island, which contains a complex of wetlands with both tidal and freshwater influences, had tidal flood gates prior to the 1990s that were later removed, leading to the expansion of mangroves, after which shorebird populations declined significantly; mangrove removal since 2016 with the goal of restoring shorebird habitat has led to an increase in shorebird populations since that time (Reid 2019). In addition, Stockton Sandspit, an artificially created landscape formed from dredge spoil that provides roosting habitat for about a quarter of the region's shorebirds, now

comprises largely natural habitats but requires regular mangrove and weed removal to retain its suitability for shorebird roosting (NSW National Parks & Wildlife Service, 2015).

Inner Deep Bay, Hong Kong (also known as Shenzhen Bay)

The intertidal wetland in the Inner Deep Bay is an Important Bird Area and Ramsar site (22.50°N, 114.02°E), supporting 36,000 – 44,000 migratory shorebirds annually, including the Spoon-billed Sandpiper *Calidris pygmaea* (CR), Spotted Greenshank *Tringa guttifer* (EN) and Asian Dowitcher *Limnodromus semipalmatus* (NT) (BirdLife International 2005; Ramsar 2014; Anon 2016). The area of mangroves in this site increased by more than 4-fold from 1.24 to 5.07 km^2 between 1973 and 2015 (Jia et al. 2016), replacing the bare tidal flats that serve as important foraging and roosting sites to migratory waterbirds (Figure 2b). The Hong Kong government alone has spent 12,900 USD annually since 2002 (about 3,355 USD per km^2) to control the spread of mangroves and maintain adequate bare intertidal flat for waterbirds (K.-S. Leung *pers. comm.*).

Guandu, Taiwan

Guandu (25.115°N, 121.47°E) is part of an Important Bird Area located at 10 km upstream from the estuary of Tamsui River in northern Taiwan (Figure 2c). The estuarine wetlands around Guandu support over 3,000 shorebirds annually. To prevent habitat destruction caused by human activities, the northern side of the river was designated as the Guandu Nature Park (0.57 km^2) and the Guandu Nature Reserve (0.55 km^2). The former has been actively managed to preserve saltmarsh habitats while the latter, strictly prohibiting any human disturbances. Since the establishment of Guandu Nature Reserve in 1986, the *Kandelia* mangrove expanded its extent from 8.7% to 70% of the Guandu area within two decades (Hsu & Lee 2010), replacing most of the reed marshes and bare intertidal flats (Lee et al. 2002), making Guandu Nature Reserve no longer suitable for its primary conservation target – waterfowls and shorebirds. After 2000, the core habitat for shorebirds shifted from the north to the south of the river, coincided with the mangrove expansion in the north and new or restored bare intertidal flats formed in the south, but the latter was also threatened

by mangrove expansion (Wild Bird Society of Taipei 1995; Huang et al. 2012; Lai 2013). In 2018, Taipei City Government proposed to delist Guandu Nature Reserve under Cultural Heritage Preservation Act and manage the area according to the less strict Wetland Conservation Act to control the spread of mangrove and conserve waterbird habitats. This proposal remains controversial as some opponents disagree with mangrove deforestation while others worry that this will set the first example of nature reserve delisting and cause more reserves to follow given the strong pressure of land development.

Leizhou Peninsula, mainland China

The Leizhou Peninsula (20.96°N, 109.97°E) is located in the south of China and has a coastline dominated by mangroves. Much of the coastal mangrove area is listed as a Ramsar site and National Nature Reserve. The intertidal wetlands in the region is important non-breeding habitat for migratory waterbirds, including 28 – 43 individuals, or 4 – 6% of the world's Critically Endangered Spoon-billed Sandpiper *Calidris pygmaea* population (Zou et al. 2006; Clark et al. 2016; Peng et al. 2017; Spoon-billed Sandpiper Conservation Alliance 2020). On the other hand, the peninsula is a strong-hold for mangroves in mainland China, comprising nearly one-third of total mangrove area in the country (Gao et al. 2009). Like many other mangroves in the world, the mangrove area in the peninsula once reached 140 km^2 in the 1950s but vanished to 58 km^2 in 1980s due to exploitation for timber and conversion into farmland and aquaculture ponds. The mangrove area bounced back to 92 km^2 in 2007 due to restoration effort between 1985 and 2007, when 22 km^2 of mangrove was planted, representing a net increase of 27% mangrove area within 22 years (Gao et al. 2009). A similar recovery (net increase of 29% between 2010 and 2015) was also found within the Zhanjiang Mangrove National Nature Reserve in Leizhou (Jia et al. 2018). Under the latest national mangrove conservation and restoration action plan, 80 km^2 of mangrove area is planned to be restored / afforested within Guangdong province by 2025 and the Leizhou Peninsula is a prime candidate for this exercise, (The Ministry of Natural Resources and the National Forestry and Grassland Administration 2020; The State Council of the People's Republic of China 2020). This plan would lead to the spread of mangroves towards bare tidal flats, demonstrating the conservation dilemma discussed.

References

Anon. 2016. Mai Po Inner Deep Bay Ramsar Site Waterbird Monitoring Programme 2015-16: Shorebird Monitoring Report.

Bai Q, et al. 2015. Identification of coastal wetlands of international importance for waterbirds: a review of China Coastal Waterbird Surveys 2005–2013. Avian Research **6**:1-16.

Bamford M, Watkins D, Bancroft W, Tischler G, Wahl J 2008. Migratory Shorebirds of the East Asian-Australasian Flyway; Population Estimates and Internationally Important Sites. Wetlands International - Oceania. Canberra, Australia.

BirdLife International. 2005. Important Bird Areas and potential Ramsar Sites in Asia. International B, Cambridge, UK.

Choi C-Y, Li J, Xue WJ. 2020. China Coastal Waterbird Census Report (Jan. 2012–Dec. 2019). Hong Kong Bird Watching Society, Hong Kong.

Clark NA, Anderson GQA, Li J, Syroechkovski EE, Tomkovich PS, Zockler C, Lee R, Green RE. 2016. First formal estimate of the world population of the Critically Endangered spoon-billed sandpiper *Calidris pygmaea*. Oryx.

Conklin JR, Verkuil YI, Smith BR. 2014. Prioritizing migratory shorebirds for conservation action on the East Asian-Australasian Flyway. Kong W-H, Hong Kong.

Forest & Bird. 2016. New Zealand Seabirds: Sites on Land, Rivers, estuaries, coastal lagoons & harbours. Wellington, New Zealand.

Gao X-m, Han W-d, Liu S-q. 2009. The mangrove and its conservation in Leizhou Peninsula, China. Journal of Forestry Research **20**:174-178.

Hsu L-C, Lee C-T. 2010. Mangrove distribution change in Kuantu Nature Reserve from 1978 to 2006. Hwa Kang Geographical Journal **26**:31-41.

Huang S-C, Shih S-S, Ho Y-S, Chen C-P, Hsieh H-L. 2012. Restoration of Shorebird-Roosting Mudflats by Partial Removal of Estuarine Mangroves in Northern Taiwan. Restoration Ecology **20**:76-84.

Jia M, Liu M, Wang Z, Mao D, Ren C, Cui H. 2016. Evaluating the Effectiveness of Conservation on Mangroves: A Remote Sensing-Based Comparison for Two Adjacent Protected Areas in Shenzhen and Hong Kong, China. Remote Sensing **8**:627.

Jia M, Wang Z, Zhang Y, Mao D, Wang C. 2018. Monitoring loss and recovery of mangrove forests during 42 years: The achievements of mangrove conservation in China. International Journal of Applied Earth Observation and Geoinformation **73**:535-545.

Lai Y-C. 2013. Long-term population trends of the wintering waterbirds at Tamsui River, Taiwan. Institute of Ecology and Evolutionary Biology, Taiwan University, Taiwan.

Lee C-T, Cheng Y-P, Chiou W-L, Lin T-T, Chen C-W, Wang I-C. 2002. Vegetation changes at the Kuantu Nature Reserve duing 1986-1998. Taiwan Journal of Forest Science **17**:41-50.

Peng H-B, Choi C-Y, Zhang L, Gan X, Liu W-L, Li J, Chiang C-Y, Wang S-L, Ma Z-J. 2017. Distribution and major threats to Spoon-billed Sandpipers in China. Chinese Journal of Zoology **52**:158-166.

Ramsar. 2014. The list of wetlands of international importance.

Reid P. 2019. Changes in wetland use by shorebirds following mangrove removal, Area E, Ash Island, New South Wales. The Whistler **13**:62-68.

Spoon-billed Sandpiper Conservation Alliance. 2020. China Spoon-billed Sandpiper Census 2020.

Swales A, Bell RG, Ovenden R, Hart C, Horrocks M, Hermansphan N, Smith RK. 2007. Mangrove habitat expansion in the southern Firth of Thames: sedimentation processes and coastal-hazards mitigation Environment Waikato Technical Report No 2008/13. Council) EWWR, Hamilton, New Zealand.

The Ministry of Natural Resources and the National Forestry and Grassland Administration. 2020. Mangrove Conservation and Restoration Special Action Plan (2020-2025), Available from

http://www.forestry.gov.cn/main/5461/20200828/145119168640816.html (accessed 11-Jan-2021).
The State Council of the People's Republic of China. 2020. *China pledges more funds for coastal ecology protection*, Available from http://english.www.gov.cn/statecouncil/ministries/202004/16/content_WS5e985951c6d0b3f0e9495962.html (accessed 11-Jan-2021).
Wild Bird Society of Taipei. 1995. Wetland birds studies on the Tamsui River (III, IV). Taipei City, Taiwan.
Zou F, Yang Q, Dahmer T, Cai J, Zhang W. 2006. Habitat Use of Waterbirds in Coastal Wetland on Leizhou Peninsula, China. Waterbirds **29**:459-464.

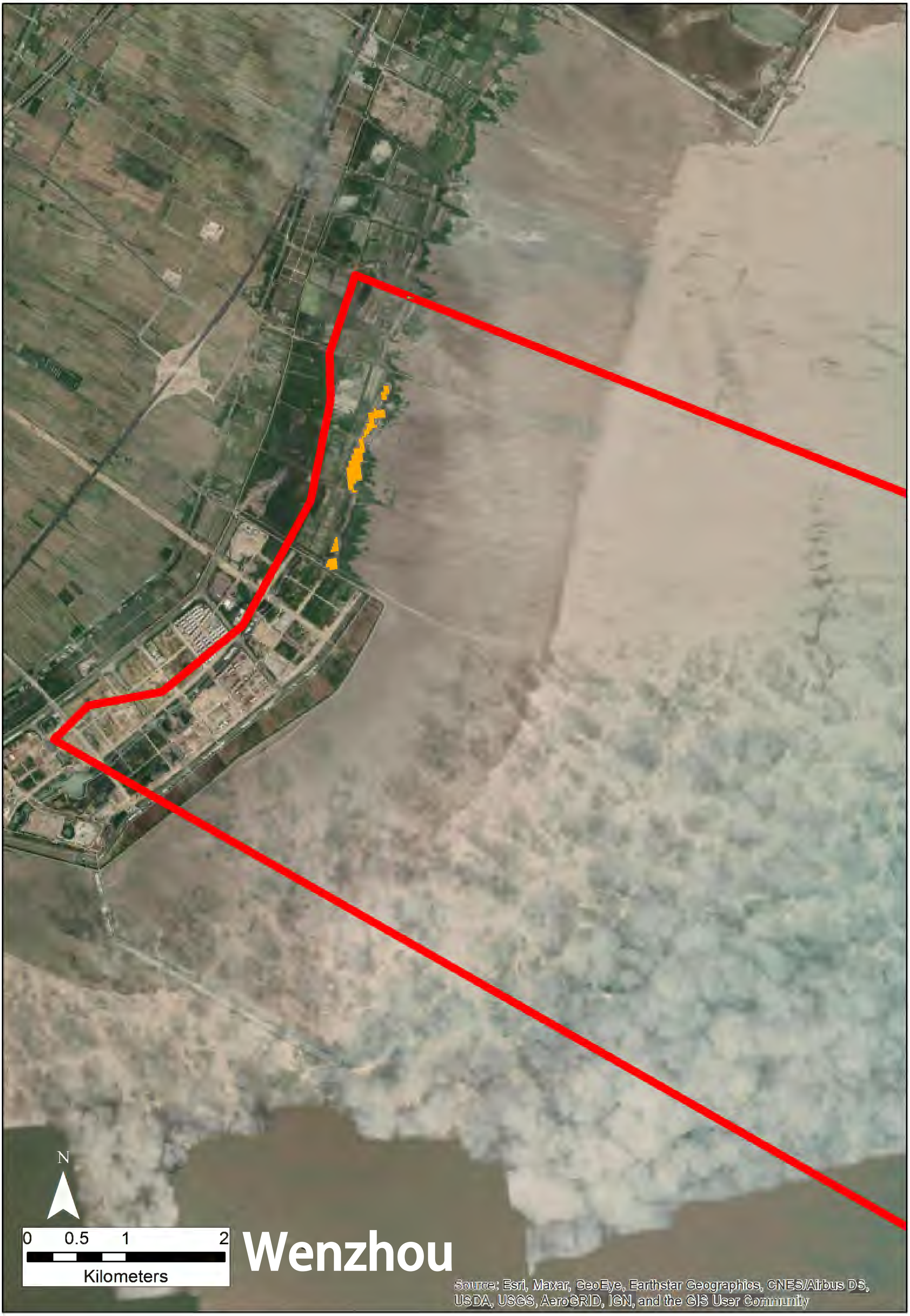
N
0 0.5 1 2
Kilometers
Wenzhou
Source: Esri, Maxar, GeoEye, Earthstar Geographics, CNES/Airbus DS, USDA, USGS, AeroGRID, IGN, and the GIS User Community

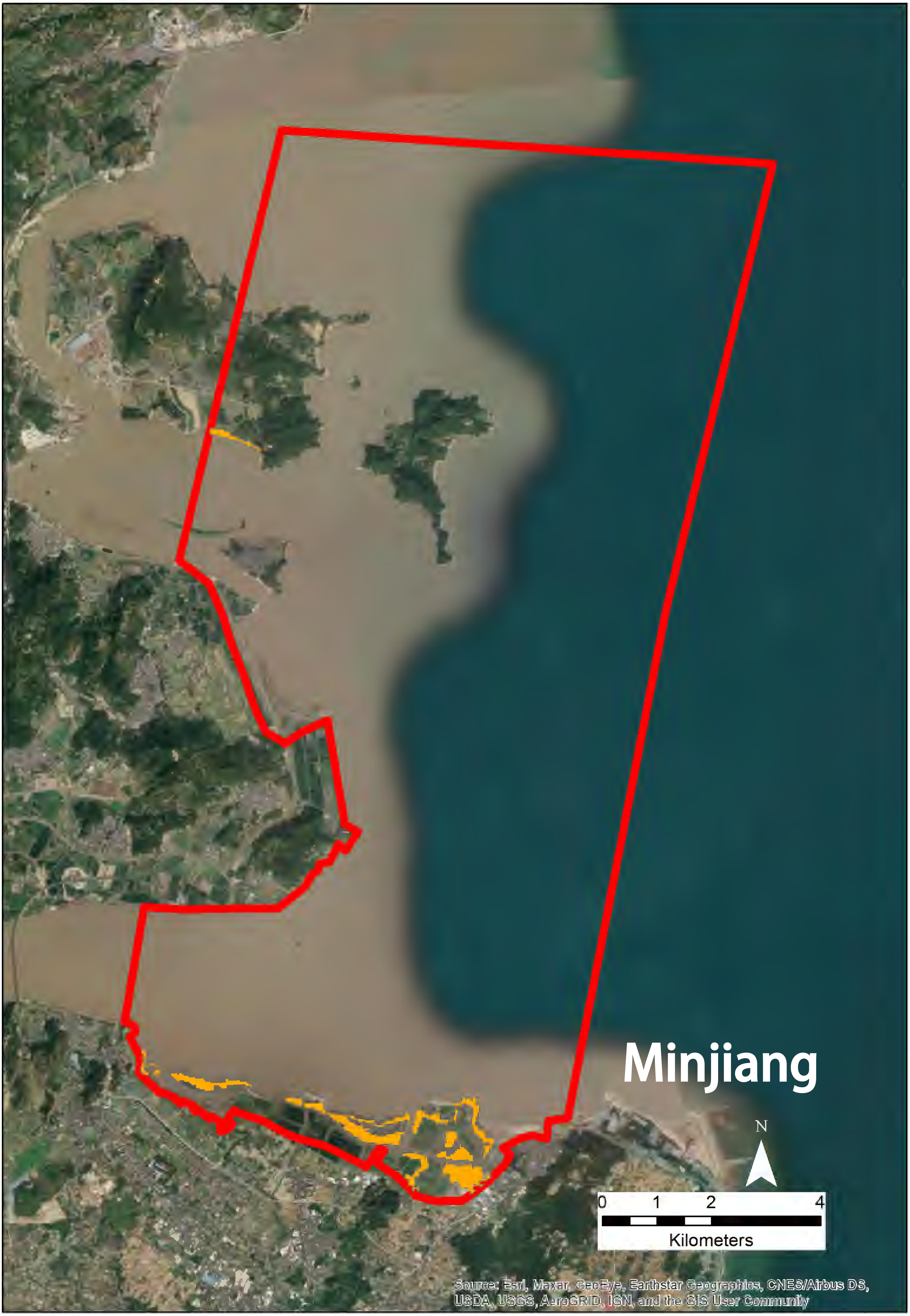
Minjiang
N
0 1 2 4
Kilometers
Source: Esri, Maxar, GeoEye, Earthstar Geographics, CNES/Airbus DS, USDA, USGS, AeroGRID, IGN, and the GIS User Community

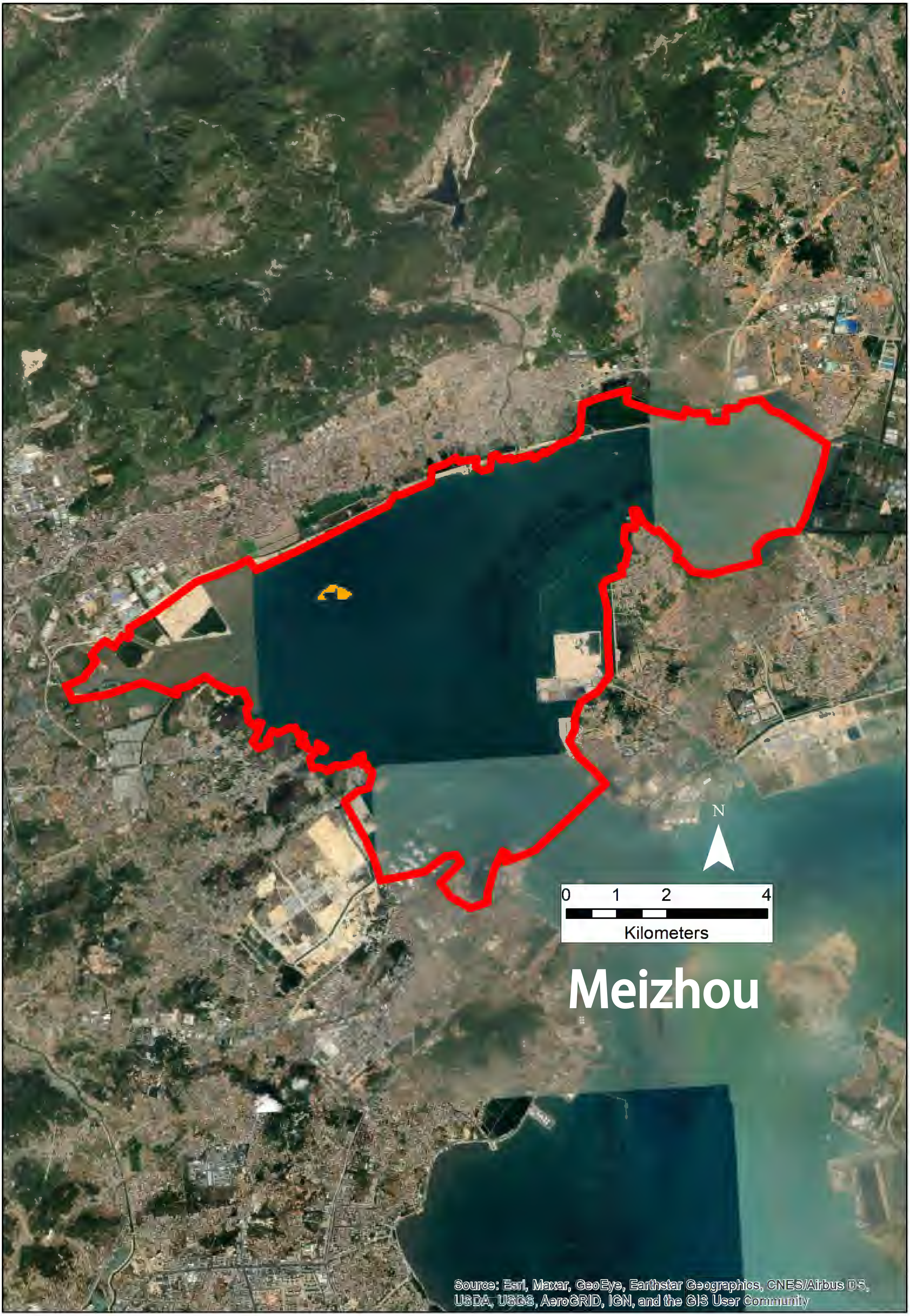
N
0 1 2 4
Kilometers
Meizhou
Source: Esri, Maxar, GeoEye, Earthstar Geographics, CNES/Airbus DS, USDA, USGS, AeroGRID, IGN, and the GIS User Community

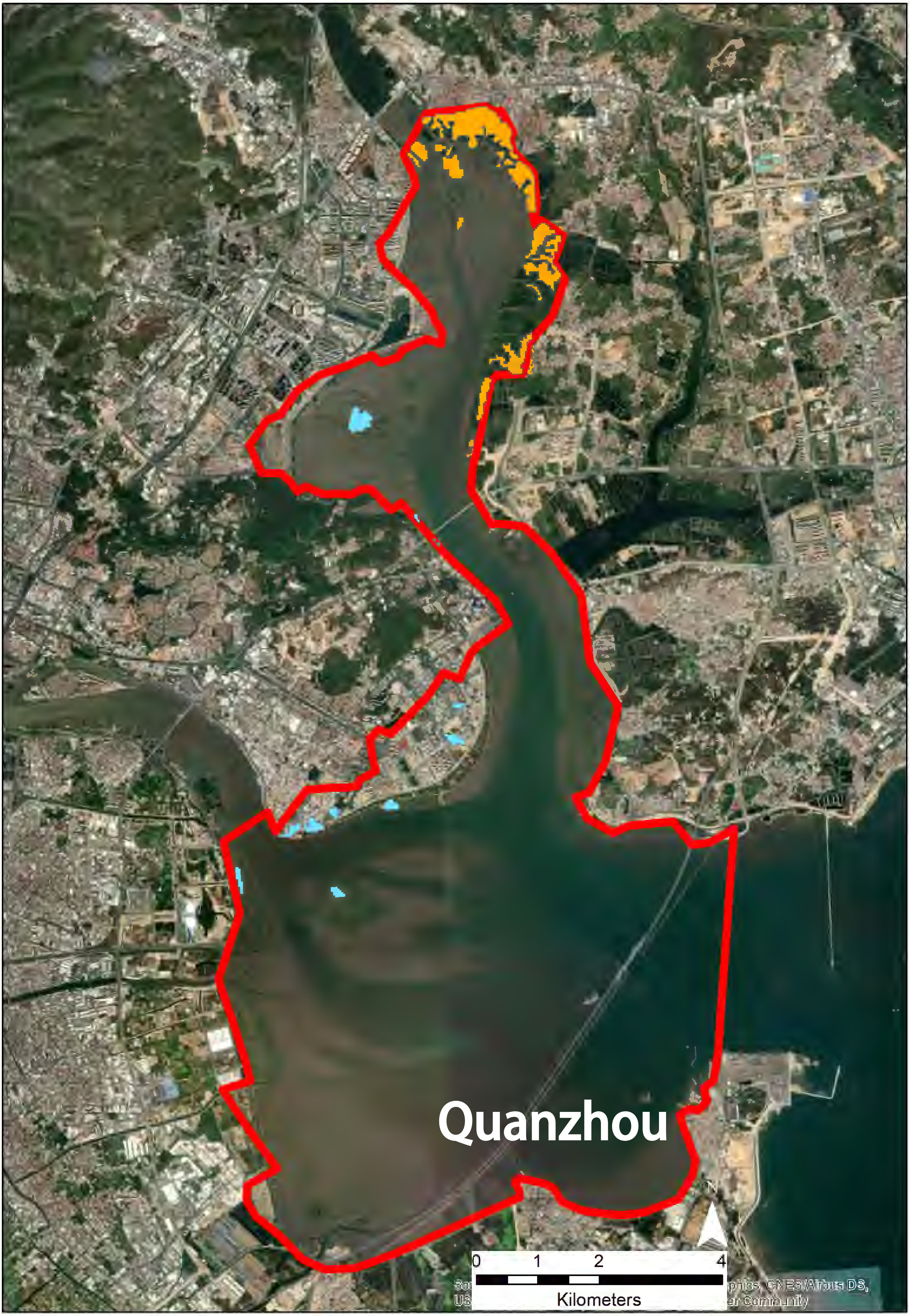
Quanzhou
0 1 2 4
Kilometers

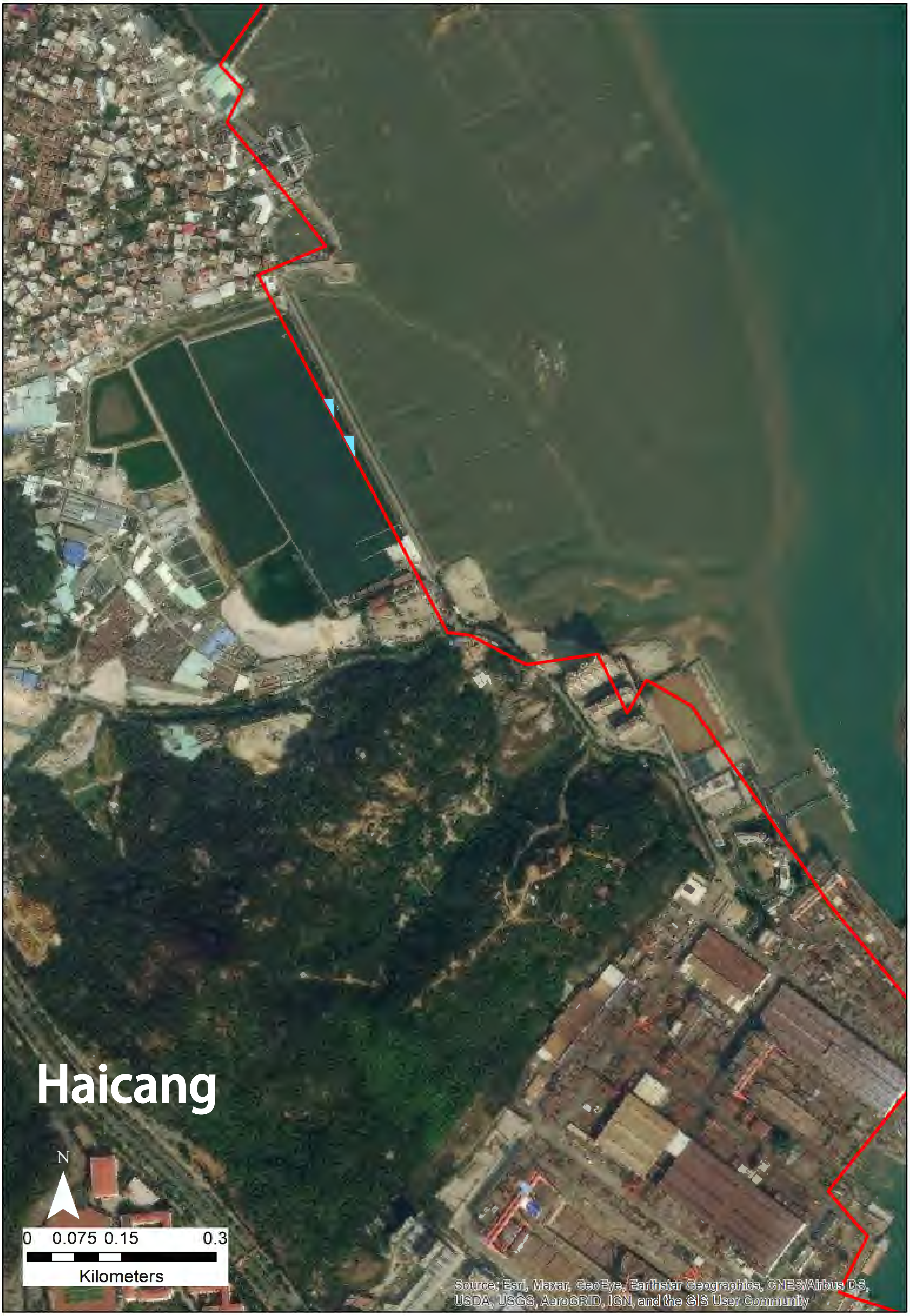

Haicang
N
0 0.075 0.15 0.3
Kilometers
Source: Esri, Maxar, GeoEye, Earthstar Geographics, CNES/Airbus DS, USDA, USGS, AeroGRID, IGN, and the GIS User Community

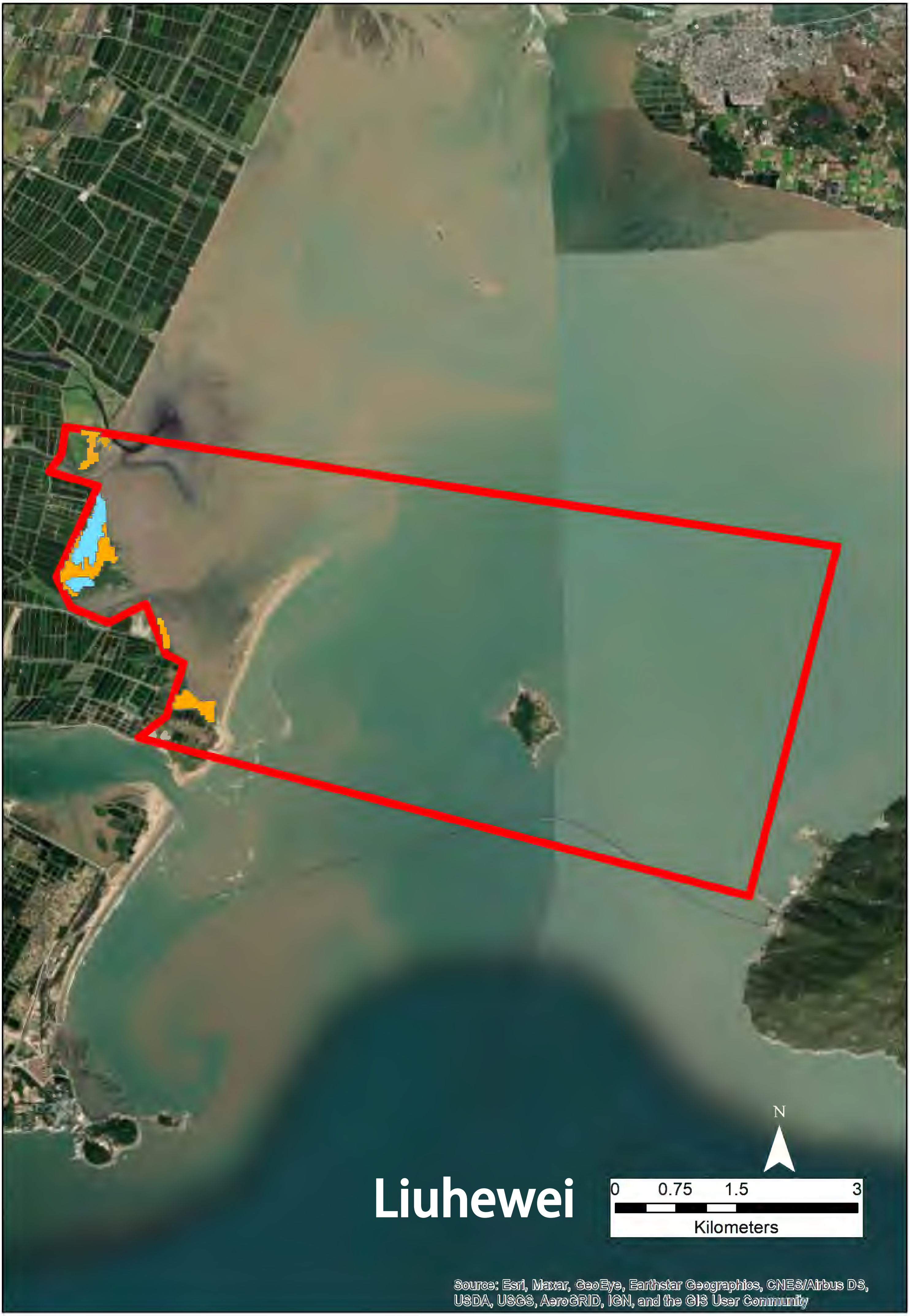
Liuhewei
N
0 0.75 1.5 3
Kilometers
Source: Esri, Maxar, GeoEye, Earthstar Geographics, CNES/Airbus DS, USDA, USGS, AeroGRID, IGN, and the GIS User Community

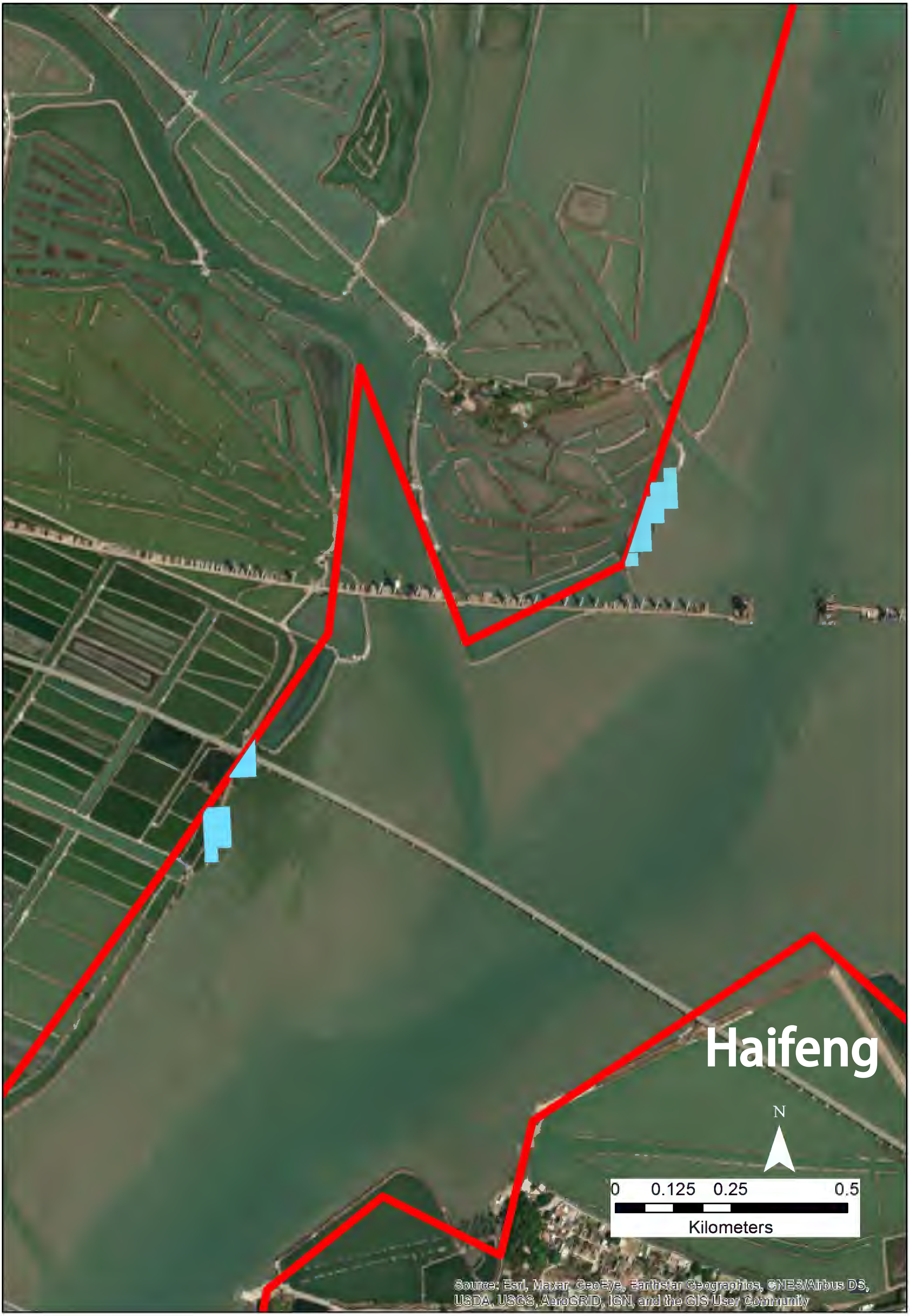
Haifeng
N
0 0.125 0.25 0.5
Kilometers
Source: Esri, Maxar, GeoEye, Earthstar Geographics, CNES/Airbus DS, USDA, USGS, AeroGRID, IGN, and the GIS User Community

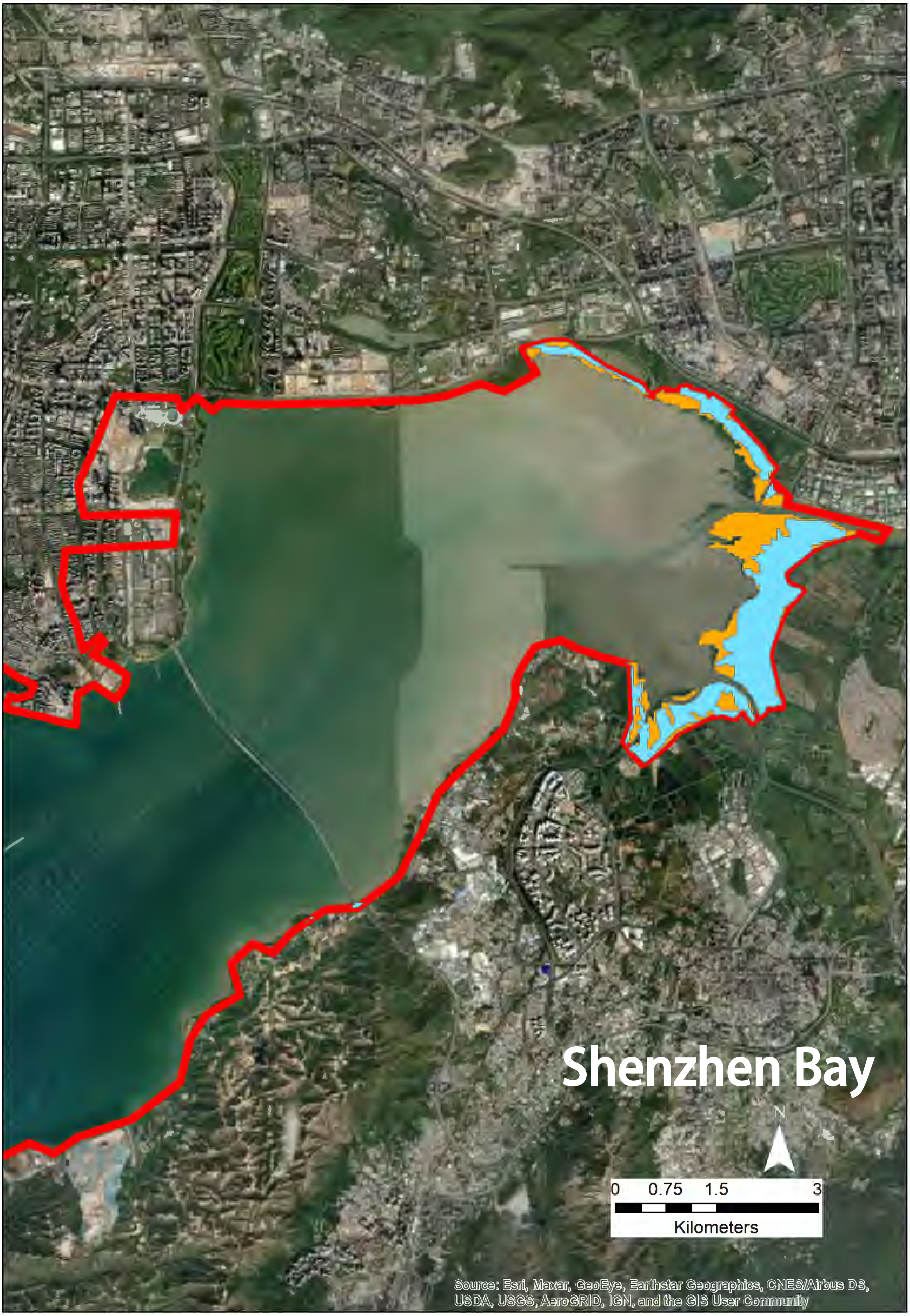
Shenzhen Bay
N
0 0.75 1.5 3
Kilometers
Source: Esri, Maxar, GeoEye, Earthstar Geographics, CNES/Airbus DS, USDA, USGS, AeroGRID, IGN, and the GIS User Community

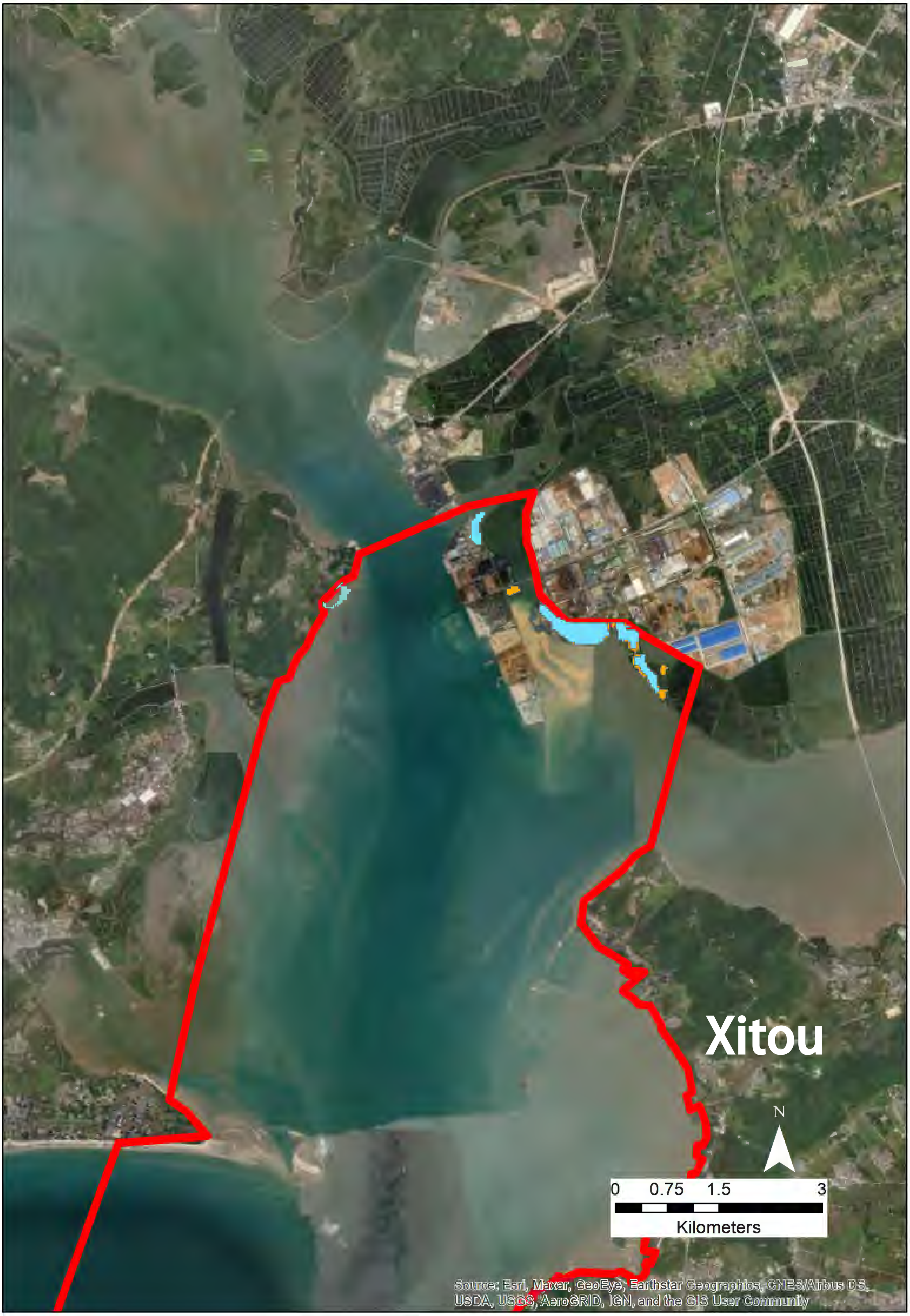
Xitou
N
0 0.75 1.5 3
Kilometers
Source: Esri, Maxar, GeoEye, Earthstar Geographics, CNES/Airbus DS, USDA, USGS, AeroGRID, IGN, and the GIS User Community

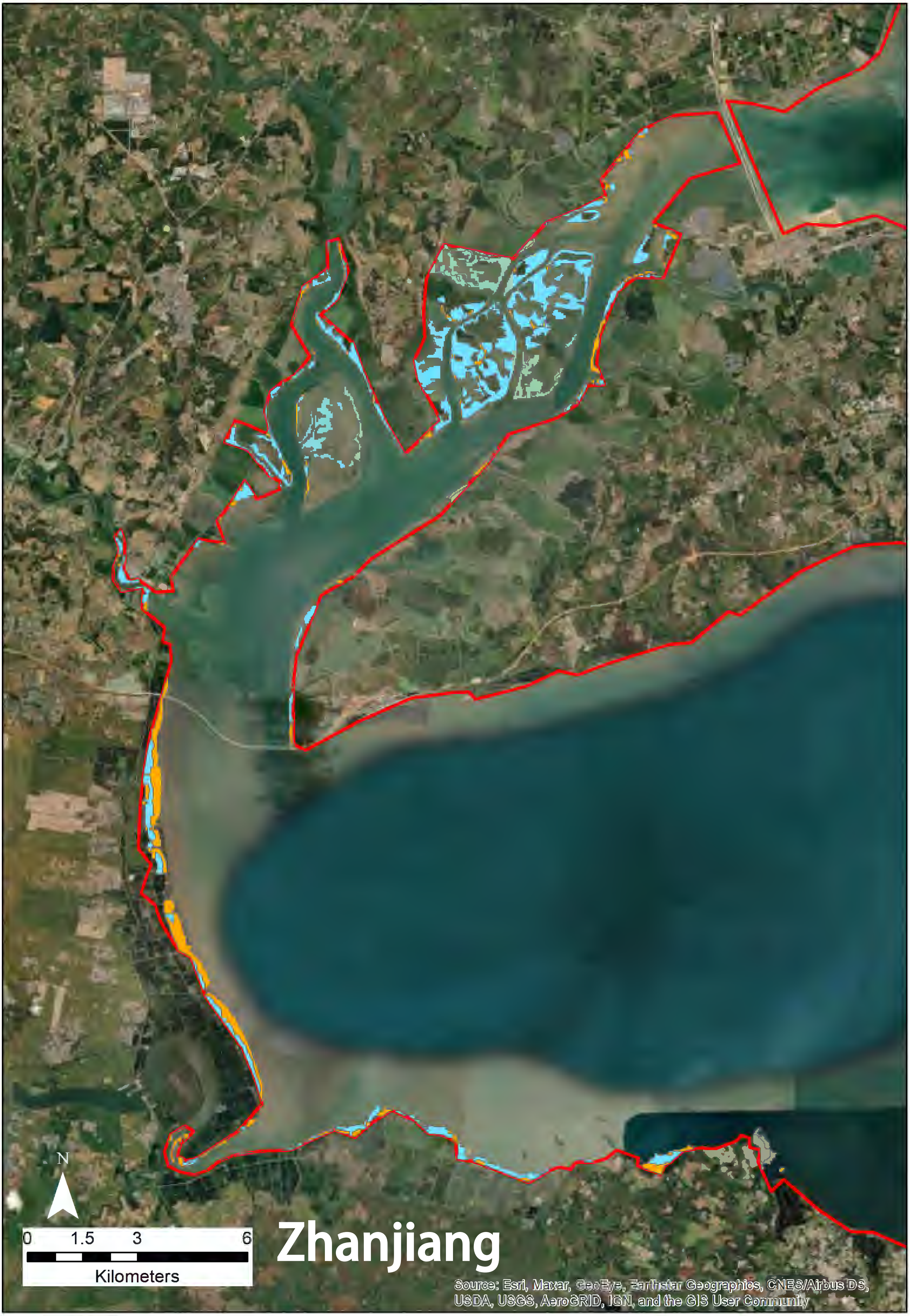
N
0 1.5 3 6
Kilometers
Zhanjiang
Source: Esri, Maxar, GeoEye, Earthstar Geographics, CNES/Airbus DS, USDA, USGS, AeroGRID, IGN, and the GIS User Community

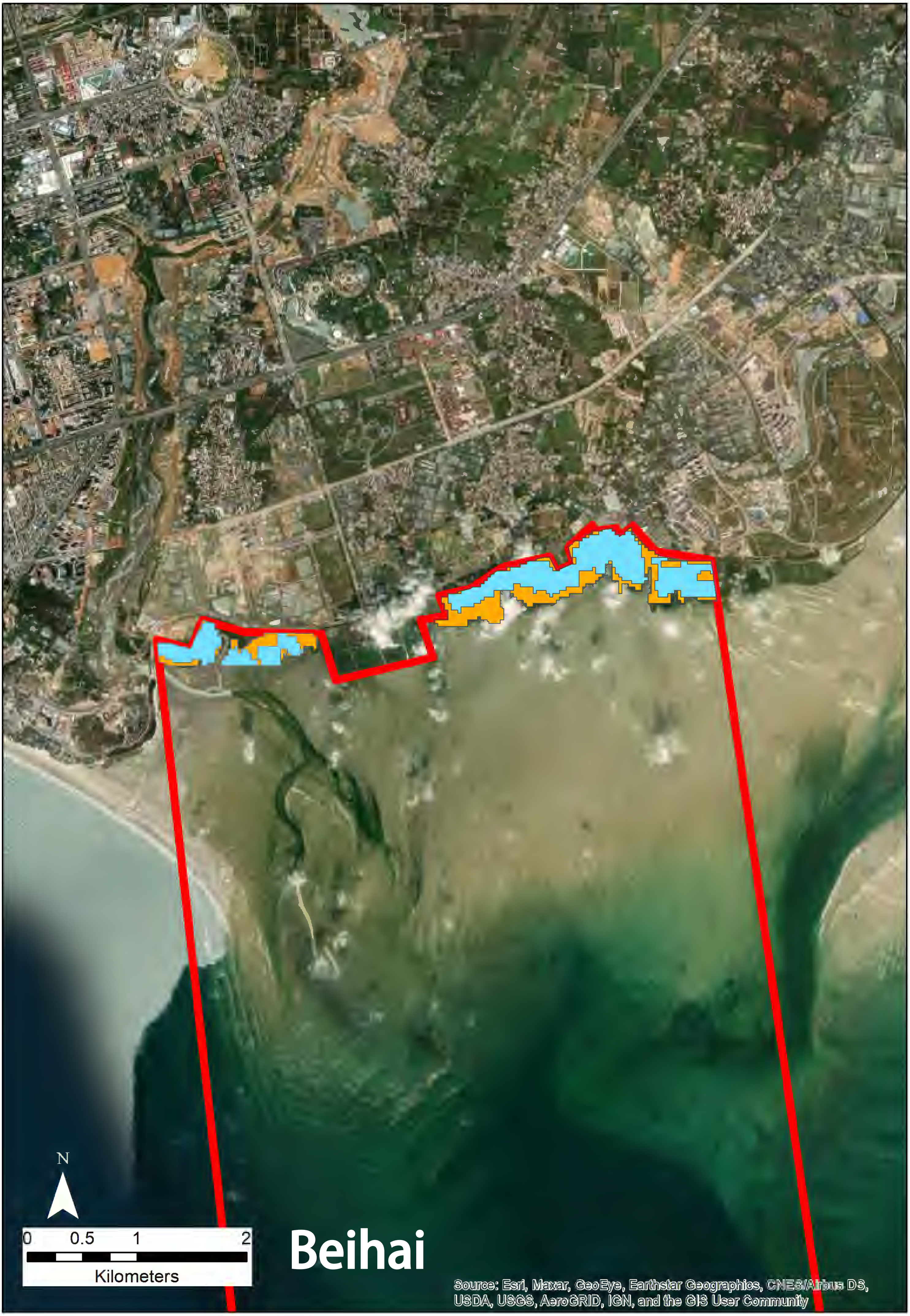
N
0 0.5 1 2
Kilometers
Beihai
Source: Esri, Maxar, GeoEye, Earthstar Geographics, CNES/Airbus DS, USDA, USGS, AeroGRID, IGN, and the GIS User Community

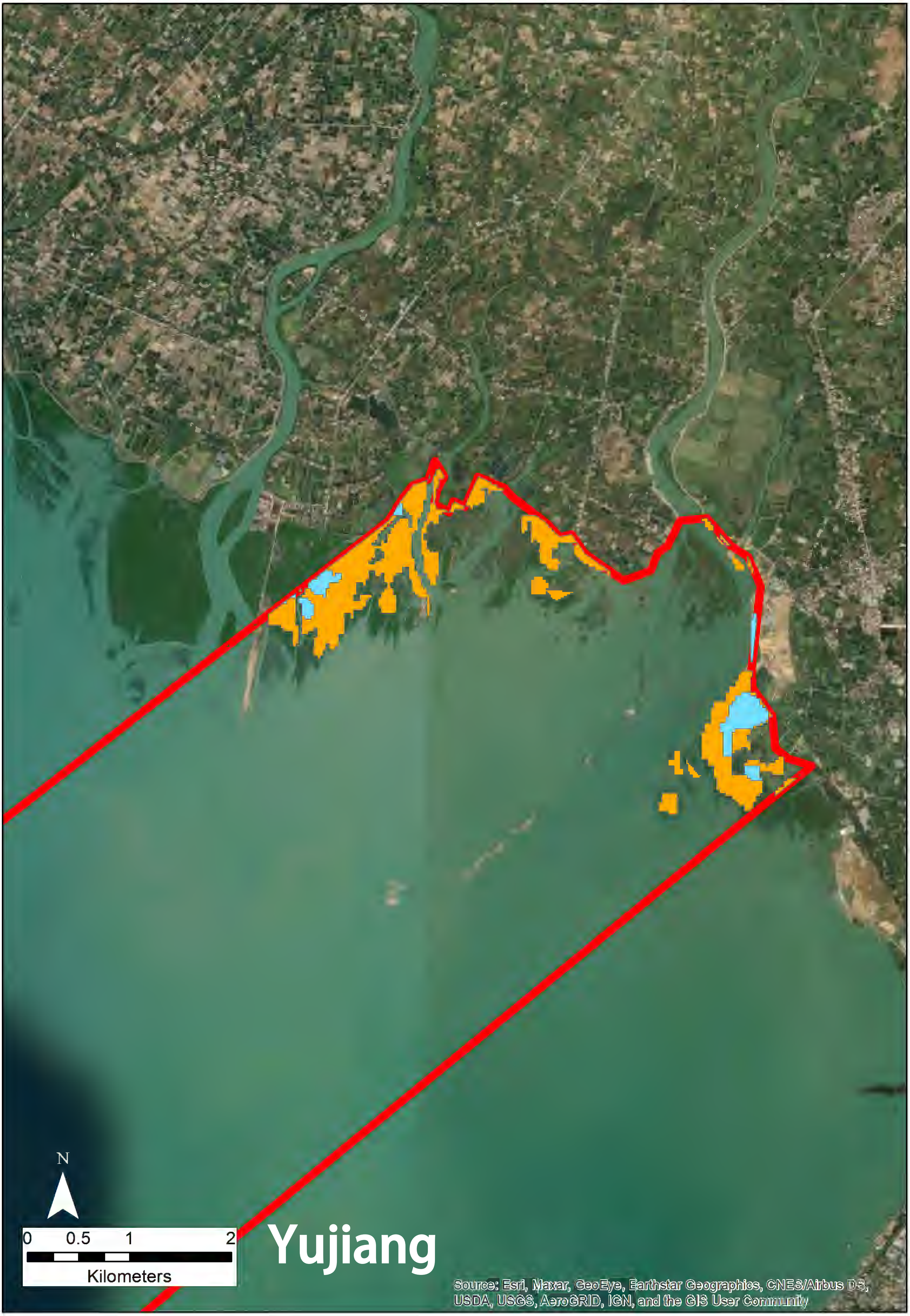
N
0 0.5 1 2
Kilometers
Yujiang
Source: Esri, Maxar, GeoEye, Earthstar Geographics, CNES/Airbus DS, USDA, USGS, AeroGRID, IGN, and the GIS User Community

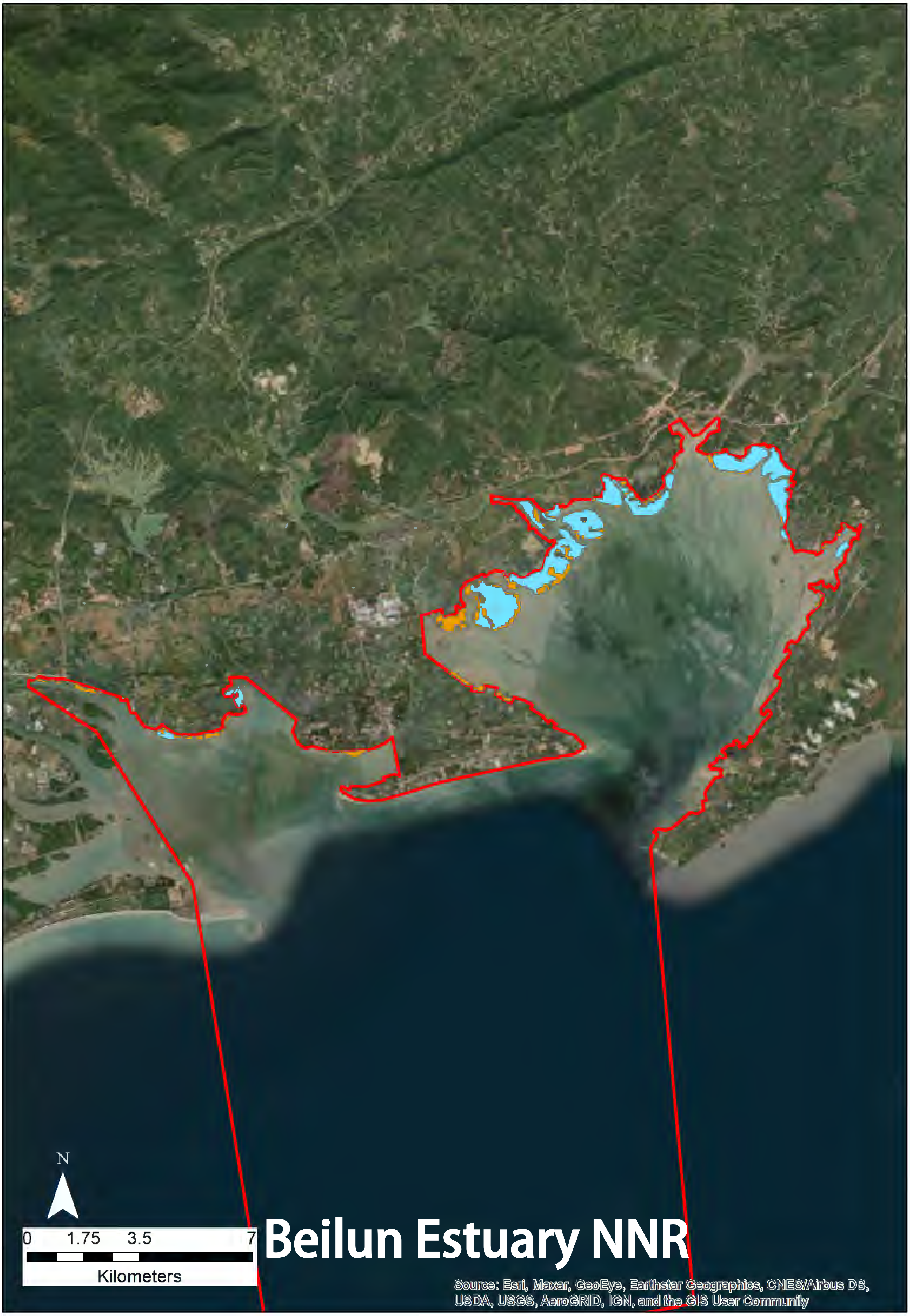
N
0 1.75 3.5 7
Kilometers
Beilun Estuary NNR
Source: Esri, Maxar, GeoEye, Earthstar Geographics, CNES/Airbus DS, USDA, USGS, AeroGRID, IGN, and the GIS User Community

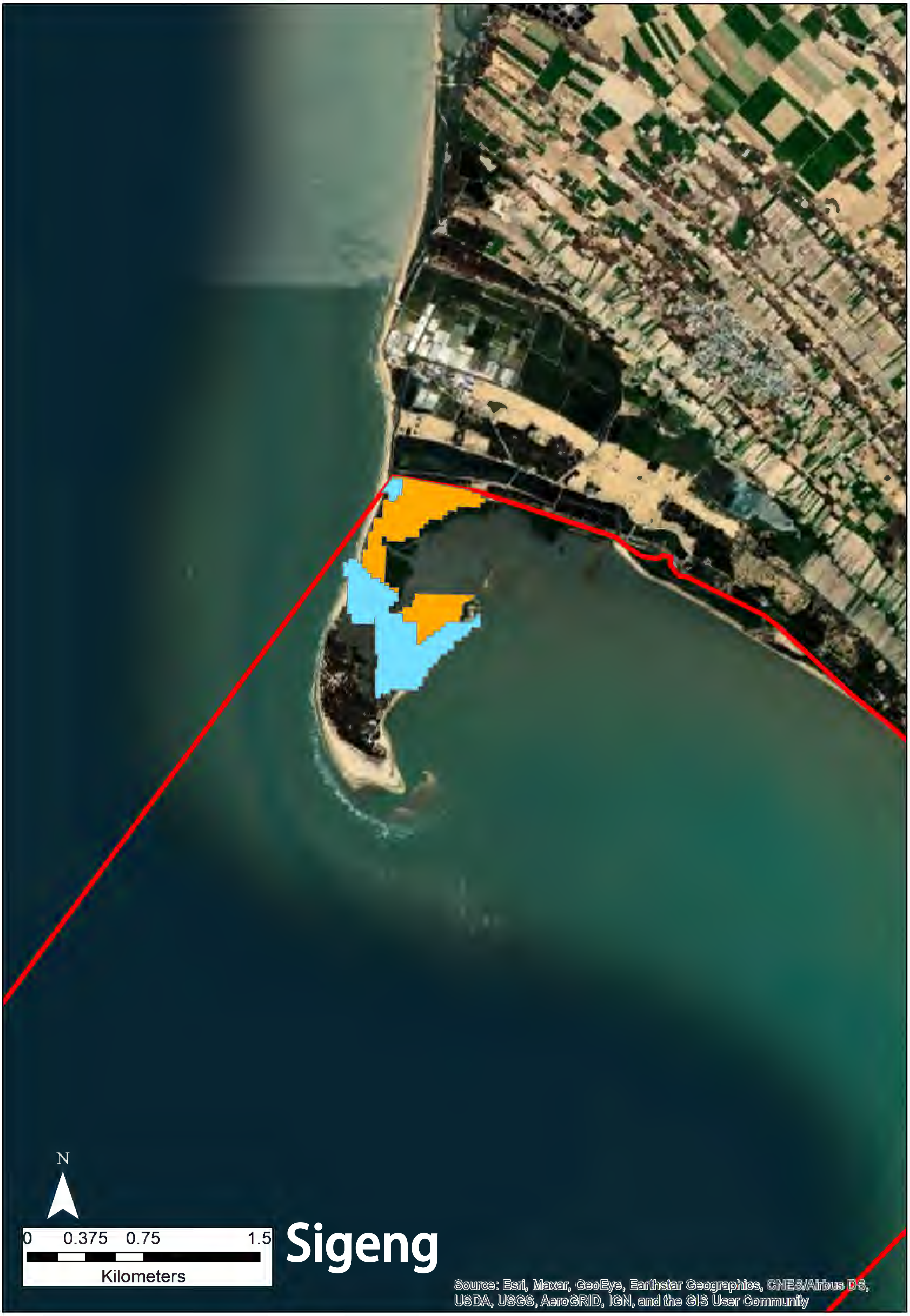
N
0 0.375 0.75 1.5
Kilometers
Sigeng
Source: Esri, Maxar, GeoEye, Earthstar Geographics, CNES/Airbus DS, USDA, USGS, AeroGRID, IGN, and the GIS User Community
